# Iridium-catalyzed hydroacylation reactions of C1-substituted oxabenzonorbornadienes with salicylaldehyde: an experimental and computational study

**DOI:** 10.3762/bjoc.18.30

**Published:** 2022-03-02

**Authors:** Angel Ho, Austin Pounder, Krish Valluru, Leanne D Chen, William Tam

**Affiliations:** 1 Guelph-Waterloo Centre for Graduate Work in Chemistry and Biochemistry, Department of Chemistry, University of Guelph, Guelph, Ontario, N1G 2W1, Canada

**Keywords:** C–H activation, density functional theory, hydroacylation, iridium catalysis, regioselectivity

## Abstract

An experimental and theoretical investigation on the iridium-catalyzed hydroacylation of C_1_-substituted oxabenzonorbornadienes with salicylaldehyde is reported. Utilizing commercially available [Ir(COD)Cl]_2_ in the presence of 5 M KOH in dioxane at 65 °C, provided a variety of hydroacylated bicyclic adducts in up to a 95% yield with complete stereo- and regioselectivity. The mechanism and origins of selectivity in the iridium-catalyzed hydroacylation reaction has been examined at the M06/Def2TZVP level of theory. The catalytic cycle consists of three key steps including oxidative addition into the aldehyde C–H bond, insertion of the olefin into the iridium hydride, and C–C bond-forming reductive elimination. Computational results indicate the origin of regioselectivity is involved in the reductive elimination step.

## Introduction

Organic synthesis is the art and science of selective molecular engineering [1]. To date, organic synthesis has largely been governed by the interconversion of pre-existing functional groups through the use of more traditional transition-metal-catalyzed cross-coupling reactions [2–5]. Although these reactions have revolutionized the modern chemist’s synthetic toolbox, prior installation of these functional groups requires a number of steps, leading to undesired side-products and reduced overall yield. An attractive alternative is the catalytic activation and subsequent functionalization of otherwise inert carbon–hydrogen bonds [6–13]. Hydroacylation reactions, the formal addition of an aldehyde C–H bond across a C–C π-system, has emerged as a powerful, and highly atom-economic approach to synthesize ketones. As such, C–H functionalizations are inherently both environmentally benign and economically attractive.

**Scheme 1:**
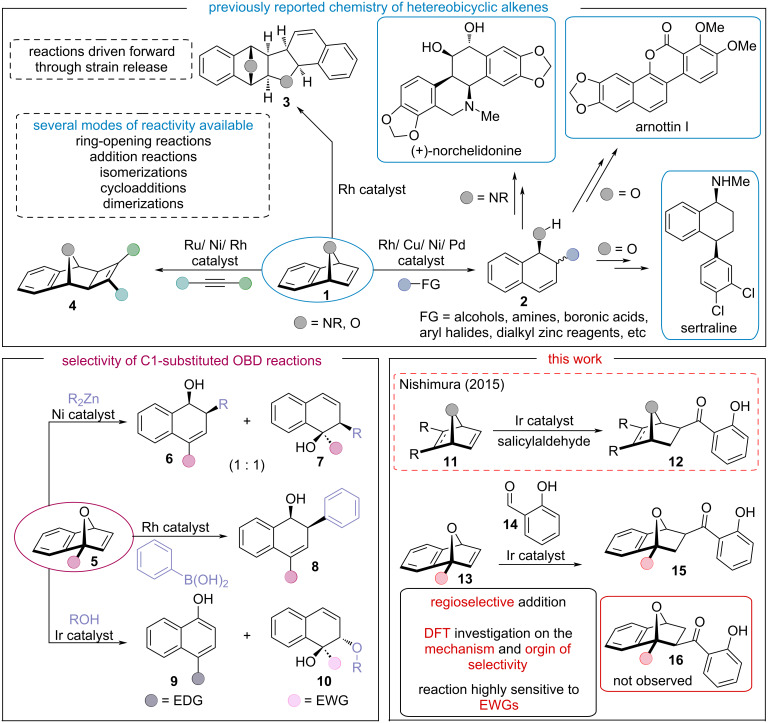
Previously reported metal-catalyzed reactions of heterobicyclic alkenes and applications towards the synthesis of biologically active compounds (top). Representative examples of regioselectivity in different metal-catalyzed ring-opening reactions of C_1_-substituted oxabenzonorbornadiene derivatives (bottom left). Nishimura’s seminal report on iridium-catalyzed hydroacylation reactions of bicyclic alkenes and the context of this work.

Transition-metal-catalyzed reactions of strained bicyclic derivatives have been an intense area of research in the last 20 years (Scheme 1) [14–17]. Of particular interest is oxabenzonorbornadiene (OBD, **1**), as it bears multiple points of reactivity that allow for diverse functionalization. Over the years, several interesting transformations have been investigated such as cycloadditions **4** [18–23], dimerizations **3** [24–27], isomerizations [28–31], among other reactions that have been reported [32–38]. The nucleophilic ring-opening reactions of heterobicyclic alkenes are of particular interest [39–53], as they provide access to a broad family of synthetic building blocks bearing multiple stereocenters in a single step **2** [54]. Application of these functionalized intermediates have found use in the total synthesis of (+)-norchelidonine (an isoquinoline alkaloid) [55], sertraline (an antidepressant) [56], and arnottin I (an anti-inflammatory) [57].

Although OBD **1** has been shown to undergo many different modes of reactivity in both a stereo- and enantioselective manner, the regioselectivity of such reactions is still undefined (Scheme 1) [58]. While the chemistry of symmetric OBD derivatives is well established, their unsymmetrically substituted counterparts **5** have remained underexplored (Scheme 1). Upon C_1_-substitution, the reactivity of C_1_-substituted OBDs **5** can greatly differ, as described by Allen and co-workers in their 2007 report on rhodium-catalyzed cyclodimerization reactions [59]. Moreover, desymmetrization of OBD produces more unique sites of reactivity allowing for the production of regioisomeric products. In 2019, Deng et al. described *syn*-stereocontrolled ring-opening reactions of oxa- and azabicyclic olefins with dialkylzinc reagents catalyzed by a nickel compound (Scheme 1) [60]. The reaction was entirely stereoselective; however, unsymmetrical OBDs **5** produced mixtures of regioisomers **6** and **7**. In the same year, Hill and co-workers published a study about the regioselective nucleophilic ring opening of C_1_-substituted OBDs **5** with water and alcohol and with an iridium compound as a catalyst (Scheme 1) [61]. Their study found the electronic nature of the C_1_-substituent controlled the regioselectivity of the reaction. Electron-donating groups (EDGs) led to naphthol compounds **9**, while electron-withdrawing groups (EWGs) led to the anticipated ring-opened 1,1,2-trisubstituted naphthalene framework **10** [61]. On the other hand, Edmunds and co-workers described a ring-opening reaction of C_1_-substituted OBDs **5** with arylboronic acids that was catalyzed by rhodium/diene to afford the 1,2,4-trisubstituted naphthalene framework **8** with complete regio- and stereocontrol (Scheme 1) [62–63].

In 2015, the Nishimura group reported the first iridium-catalyzed addition of salicylaldehydes **14** to bicyclic alkenes **11** (Scheme 1) [64]. Although a variety of carbo- and heterobicyclic alkenes was investigated, the study was limited by the number of unsymmetrical coupling partners. In their seminal report, the authors were able to produce hydroacylated adducts **15a** and **15b** in good yield. On the basis of the aforementioned literature, several different products can be formed based on a complex relationship between the reactants, C_1_-substituent, and reaction conditions [58]; therefore, it is paramount to understand of the effects that C_1_-substitution has on the reactions. Inspired by the initial work of Nishimura and co-workers [64], we pursued a study on the effects of C_1_-substitution on the iridium-catalyzed hydroacylation reactions of unsymmetrical OBDs with salicylaldehyde. To further understand the observed regioselectivity, an in-depth investigation into the reaction mechanism of the iridium-catalyzed hydroacylation reaction was carried out by preforming density functional theory calculations. We set out to confirm the catalytic cycle in detail, the geometries of the intermediates, the energy profiles of the reactions, and most importantly, the origin of regioselectivity.

## Results and Discussion

### Experimental

We began our investigation with C_1_-methyl-substituted OBD (MeOBD, **13b**) (Table 1). The use of [Ir(COD)Cl]_2_ (5 mol %) and 5 M KOH in H_2_O (10 mol %) in 1,4-dioxane at 65 °C for 20 h were the optimal conditions for the hydroacylation reaction (Table 1, entry 1) exclusively affording the C_3_-regioisomer **15b** in a 61% isolated yield. To the effect of the dummy ligand present on the active iridium species, tetrabutylammonium salts were added (Table 1, entries 4 and 5); however, these were not as efficient in the reaction. Other iridium sources (Table 1, entries 2 and 3) proved to be not as effective in promoting the reaction, with Vaska’s complex failing to react. Alternative bases (Table 1, entries 7–10) were tested; however, the reaction produced isomerized naphthol derivative **17** rather than the predicted addition product. These results indicate the formation of a phenoxoiridium(I) species assists in the oxidative addition of the C–H bond, as previously put forth by Nishimura and co-workers [64]. Isomerization of the oxabicyclic starting material to **17** may operate through a similar process described at Hill and co-workers (Scheme 1) [61]. Irreversible C–O insertion of the chloroiridium(I) species at the more electron-dense C_1_-position affords an enyliridium(III) alkoxide complex which eventually leads to the formation of isomerized 1-naphthol products [61]. Interestingly, the loading of the iridium precatalyst (Table 1, entries 11–13) also had a substantial effect on the isomerization of **13b**, with increased loading producing more byproduct. Other solvents (Table 1, entries 16–20) were explored but were not as efficient in the reaction, producing mixtures of **15b** and **17**.

**Table 1:** Optimization of the hydroacylation reaction of MeOBD **13b** with salicylaldehyde (**14**).

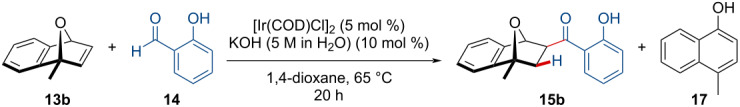			

entry	deviation from standard conditions	yield **15b** (%)^a^	yield **17** (%)^a^

1	none	61	0
2	Ir(CO)Cl(PPh_3_)_2_ instead of [Ir(COD)Cl]_2_	0	0
3	[Ir(COD)_2_]BF_4_ instead of [Ir(COD)Cl]_2_	23	0
4	10% TBAI^b^ as an additive	37	0
5	10% TBABr^c^ as an additive	28	0
7	Na_2_CO_3_ instead of KOH	45	45
8	NaOH instead of KOH	34	22
9	Cs_2_CO_3_ instead of KOH	22	49
10	no base	13	64
11	1% [Ir(COD)Cl]_2_ loading	0	0
12	10% [Ir(COD)Cl]_2_ loading	25	23
13	20% [Ir(COD)Cl]_2_ loading	19	57
14	90 °C instead of 65 °C	32	25
15	110 °C instead of 65 °C	36	34
16	DME instead of 1,4-dioxane	38	13
17	DMF instead of 1,4-dioxane	31	0
18	MeCN instead of 1,4-dioxane	23	0
19	DCE instead of 1,4-dioxane	0	61
20	THF instead of 1,4-dioxane	0	39

^a^Isolated yields. ^b^Tetrabutylammonium iodide. ^c^Tetrabutylammonium bromide.

**Scheme 2:**
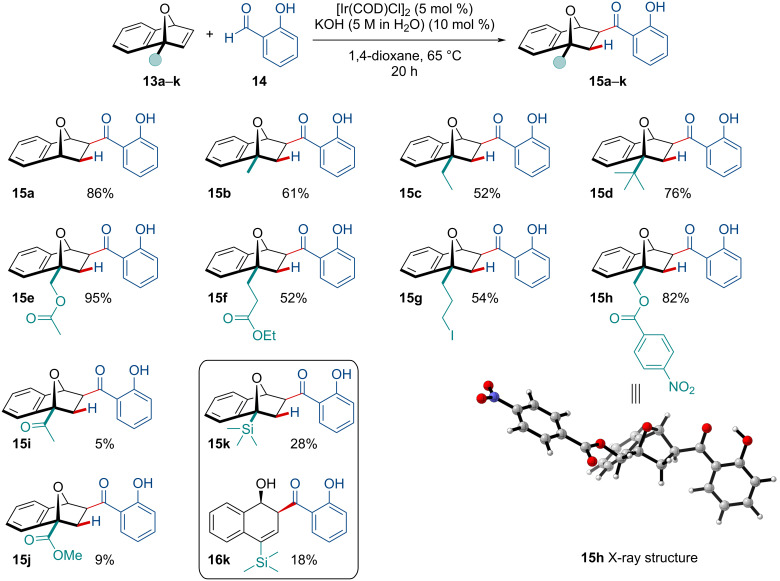
Iridium-catalyzed hydroacylation of C_1_-substituted OBDs **13a–k** with salicylaldehyde **14**.

The scope of the reaction was expanded to include different C_1_-substituted OBDs to investigate the electronic and steric effects of the C_1_ functionality on the hydroacylation reaction (Scheme 2). Satisfyingly, the reaction exclusively afforded the C_3_-hydroacylated regioisomer **15** in all cases. Moreover, the reaction was stereoselective for the formation of the *exo*-adduct rather than a mixture of *endo*/*exo* products as previously reported by Tanaka/Suemune [65] and Bolm [66], who independently studied the rhodium-catalyzed intermolecular hydroacylation reaction of salicylaldehydes with norbornadiene derivatives. It was found that electron-donating moieties at the C_1_-position were well tolerated in the hydroacylation reaction giving methyl- (**15b**), ethyl- (**15c**), and *t*-Bu- (**15d**) adducts in a 61%, 52%, and 76% yield, respectively. A significant decrease in the yield was observed for electron-withdrawing C_1_-substituted OBDs with ketone- (**15i**) and ester- (**15j**) substituted adducts only being produced in a 5% and 9% yield, respectively; however, unreacted starting material was recovered. Interestingly, C_1_-substitution with a trimethylsilyl (TMS) group resulted in the corresponding adduct **15k** as well as the ring-opened 2,4-substituted naphthol product **16k**. It was noted the insertion of a methylene unit at the C_1_-position allowed for electron-withdrawing substituents to be present in the reaction (**15e**, **15f**, **15h**). Sensitive functional groups like alkyl iodides were tolerated in the reaction, although product yields were slightly diminished. The relative stereo- and regiochemistry of the adducts was confirmed through NMR experiments and X-ray crystallography (**15h**) [67].

**Scheme 3:**
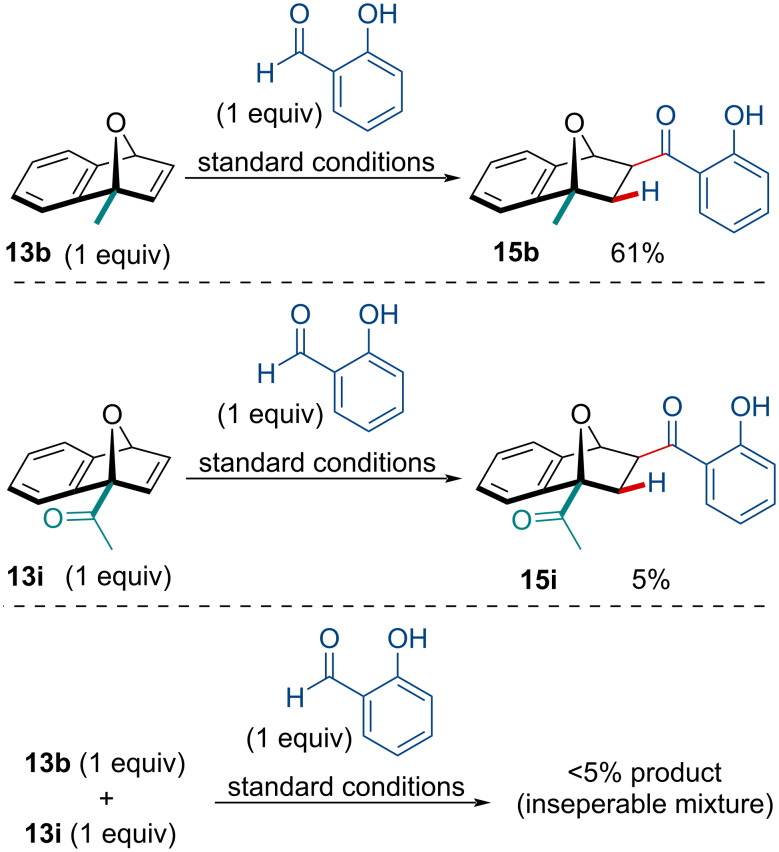
Competition reaction of different C_1_-substituted OBDs.

We next sought to determine the effect of the electron-withdrawing group on the efficacy of the reaction. Interested if the C_1_-substituted ketone OBD **13i** was merely unreactive, we subjected it to a competition reaction against C_1_-substituted methyl OBD **13b** (Scheme 3). In the presence of **13i**, the C_1_-substituted methyl OBD **13b** failed to react, giving a total <5% product yield as an inseparable mixture of both **15b** and **15i**. Noteworthy, both C_1_-substituted OBDs were recovered, with very little side-product formation. Although we are unable to confirm the precise cause of the deleterious effect, we suspect C1-substitution with electron-withdrawing groups inactivates the iridium catalyst, perhaps by chelation with the carbonyl and the bridging oxygen atom.

### Computational

#### Computational details

All density functional theory (DFT) calculations in this study were carried out with the Gaussian 16, C.01 suite of programs [68]. Geometry optimizations of all the intermediates and transition states were carried out with the Minnesota functional M06 [69] with the double-ζ basis set def2SVP [70] and Grimme’s dispersion (GD3) [71]. Harmonic vibrational frequencies were computed to verify the nature of the stationary points. The normal modes of all local minima have only real frequencies, while transition-state structures were characterized by exactly one imaginary frequency. Solvent effects (solvent = 1,4-dioxane) were taken into account using the polarized continuum model (PCM) of Tomasi and co-workers [72] and were involved in all geometry optimization and frequency calculations. Frequency analyses and single-point energies were calculated with the M06 functional [69] with the triple-ζ basis set def2TZVP [70] with the PCM (1,4-dioxane) solvent model [72]. The Gibbs free energies of formation of the reactants, products, and transition states were calculated from the optimized structures by single-point calculations by adding thermochemical corrections to the electronic energy. Optimized structures are illustrated using CYLview [73].

In order to further understand the iridium-catalyzed hydroacylation reaction, we carried out DFT calculations on the mechanism. Although an in-depth investigation into the mechanism of iridium-catalyzed hydroacylation reactions has never been carried out, the mechanistic pathways should parallel those of other analogously reactive d^7^ metals. On the basis of pioneering mechanistic investigations into rhodium-catalyzed hydroacylation reactions [74–78], we propose a catalytic cycle utilizing iridium that proceeds with three key steps: (1) iridium(I) oxidative addition into the aldehyde C–H bond, (2) insertion of the olefin into the iridium hydride, and (3) C–C bond-forming reductive elimination.

**Figure 1:**
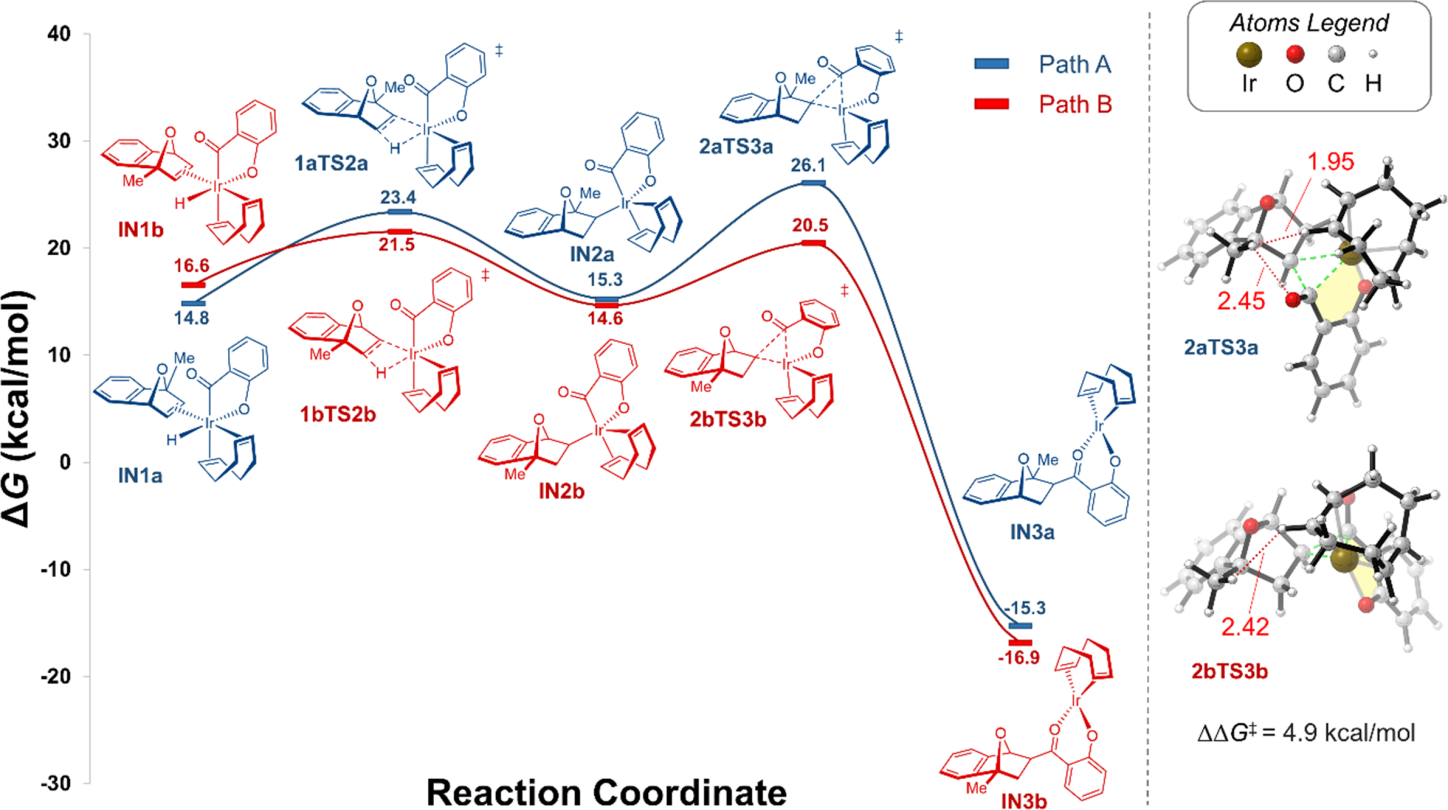
Potential energy profile of the PCM solvation model for the hydrometalation/reductive elimination pathway of the Ir/diene-catalyzed hydroacylation of MeOBD with salicylaldehyde (**14**) in 1,4-dioxane, as evaluated by DFT calculation (M06 [69]/def2SVP [70]/PCM [72]//M06 [69]/def2TZVP [70]/PCM [72]). Calculated Gibbs free energies (in kcal/mol; *T* = 338.15 K) are with respect to separated reactants Ir(COD)OH (cat) + SaliK + MeOBD. The dotted lines in the illustration of the transition states represent bonds being broken/formed. The bond lengths are given in Å.

The hydroacylation reaction with C_1_-substituted methyl OBD (MeOBD) with salicylaldehyde catalyzed by [Ir(COD)OH]_2_ was chosen as the model reaction. As the reaction is in the presence of 5 M KOH, the potassium salt of salicylaldehyde was used rather than the protonated species for all calculations. Likewise, [Ir(COD)OH]_2_, and its derivatives were used in all calculations rather than the experimentally used precatalyst [Ir(COD)Cl]_2_, as ligand exchange with the hydroxide ions present likely generates the [Ir(COD)OH]_2_ species in solution. Upon monomerization of [Ir(COD)OH]_2_, either through solvolysis or coordination of the substrate, the active catalyst Ir(COD)OH will undergo oxidative addition into the aldehyde C–H bond. Next, the iridium hydride species will undergo *exo*-η^2^-coordination with the olefin of MeOBD to generate intermediates **IN1a** and **IN1b** (Figure 1). It is typically assumed *exo*-η^2^-coordination is preferential over *endo*-η^2^-coordination, as the less congested convex face would impose reduced steric requirements. There are two potential isomeric intermediates following η^2^-coordination to the *exo*-face of MeOBD concerning the relative orientation of the COD ligand, acyl group, and C_1_-substituent on the oxabicyclic alkene. In **IN1a**, the chelated acyl group is positioned *syn* to the C_1_-methyl substituent while in **IN1b**, they are positioned *anti* to one another. **IN1b** is 1.8 kcal/mol higher in energy than its isomer which can be attributed to the increased steric interactions between the bulky COD ligand and the C_1_-methyl substituent. In both cases, the C–C’ distance of the olefin (1.40 Å) in the Ir–C–C’ coordination is marginally lengthened with respect to the separated olefin (1.33 Å) from the π back donation π*-antibonding orbital of the ligand. The next process concerns the insertion of the olefin into the iridium–hydride bond to form hydrometalated intermediates **IN2a** and **IN2b** (Figure 1). Two possible transition states, which exhibit a distorted Ir–H–C–C’ four-membered ring geometry, can be located. The concerning free energy barrier about **IN1a** to **IN2a**, via **1aTS2a** is 8.6 kcal/mol whereas it is 4.9 kcal/mol to form **IN2b**, via **1bTS2b**. Understandably, the steric interaction between the C_1_-substituent and the acyl group is responsible for this energy difference. The relative energy of the hydrometalated intermediates (**IN2a** and **IN2b**) are comparable to their preceding intermediates with **IN2a** being 0.5 kcal/mol less stable while **IN2b** is 2.0 kcal/mol more stable (Figure 1). As such, it is likely these two intermediates are strongly in equilibrium with no thermodynamic driving force favoring one over the other; moreover, the activation energies of the forward and reverse reactions are proportional.

The last key step in the catalytic cycle involves the C–C bond-forming reductive elimination to form the final ketone intermediate **IN3a** or **IN3b** (Figure 1). Two possible transition states, **2aTS3a** and **2bTS3b**, can be located. The concerning free energy barrier about **IN2a** to **IN3a**, via **2aTS3a** is 10.8 kcal/mol whereas it is 5.9 kcal/mol to form **IN3b**, via **2bTS3b**. The Ir(I) alkoxide intermediate **IN3b** (**IN3a**) is rather thermodynamically stable (Figure 1), 31.5 (30.6) kcal/mol lower in energy than the preceding intermediate **IN2b** (**IN2a**) indicating a strongly exergonic process. By comparing all the competing transition states in both reaction pathways, it is determined the reductive elimination step is the rate-determining step (RDS) for the active bond-forming hydroacylation catalytic cycle, as it possesses the greatest free energy barriers. This parallels that determined by Morehead and Sargent who hypothesized the reductive elimination step was the RDS for rhodium-catalyzed intramolecular hydroacylation reactions [74]. Based on the activation energy of the reverse step of reductive elimination **3TS2** (37.4–41.4 kcal/mol), we predict reductive elimination, and subsequent C–C formation, to be irreversible under the experimental reaction conditions. As such, we theorize the origin of regioselectivity for the title reaction is the reductive elimination step. Based on the relative kinetics, we predict the point of selectivity must occur before the irreversible C–C forming step. Comparing the two competing reductive elimination transition states, **2bTS3b** is the more energetically accessible transition state with an energy barrier of 5.9 kcal/mol, that is 4.9 kcal/mol lower in energy compared to **2aTS3a**. This large difference in activation energy (ΔΔ*G^‡^*) between the two competing transition states offers explanation towards the sole production of the experimentally observed *anti-*acylated product **15**.

**Figure 2:**
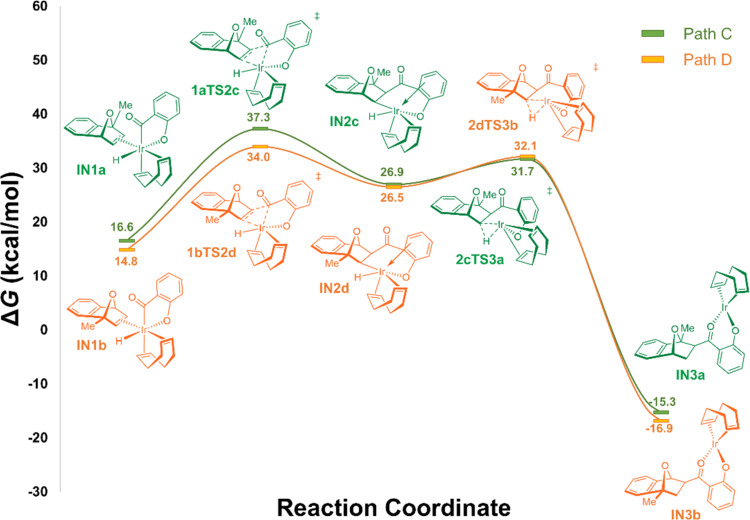
Potential energy profile of the PCM solvation model for the carbometalation/reductive elimination pathway of the Ir/diene-catalyzed hydroacylation of MeOBD with salicylaldehyde (**14**) in 1,4-dioxane, as evaluated by DFT calculation (M06 [69]/def2SVP [70]/PCM [72]//M06 [69]/def2TZVP [70]/PCM [72]). Calculated Gibbs free energies (in kcal/mol; *T* = 338.15 K) are with respect to separated reactants Ir(COD)OH (Cat) + SaliK + MeOBD.

Although Weller and co-workers have elegantly demonstrated hydride migration, rather than the alternative carbometalation, occurs during rhodium-catalyzed intermolecular alkyne hydroacylation [78], no such experiment has been carried out under iridium catalysis. As such, we set out to explore the possibility acyl migration is favored over hydride migration in iridium-catalyzed hydroacylation reactions (Figure 2). Beginning with the two *exo*-η^2^-coordinated intermediates **IN1a** and **IN1b**, two possible transition states for the acyl migration, which exhibit a distorted Ir–C–C’–C’’ four-membered ring geometry, can be located. Directly comparing the hydrometalation process (Figure 1) to the carbometalation process, we see that acyl migration is unfavored, exhibiting activation energies of 19.2 (**1bTS2d**) to 20.7 (**1aTS2c**) kcal/mol. The carbometalated intermediates **IN2c** and **IN2d** are 10.3 and 11.7 kcal/mol less stable than their preceding intermediates (**IN1a** and **IN1b**), indicating the carbometalation step is an endergonic process. Subsequent reductive elimination of the hydride ligand, via **2cTS3a** and **2dTS3b,** requires an activation energy of 4.8 to 5.6 kcal/mol, respectively, to produce the aforementioned thermodynamically stable Ir(I) alkoxide intermediates **INa3** and **INb3**. Based on the extremely high energy barrier required for acyl migration over hydride migration, we hypothesize iridium-catalyzed hydroacylation reactions proceed via the hydride migration pathway, like that reported for the rhodium-catalyzed hydroacylation reactions.

**Figure 3:**
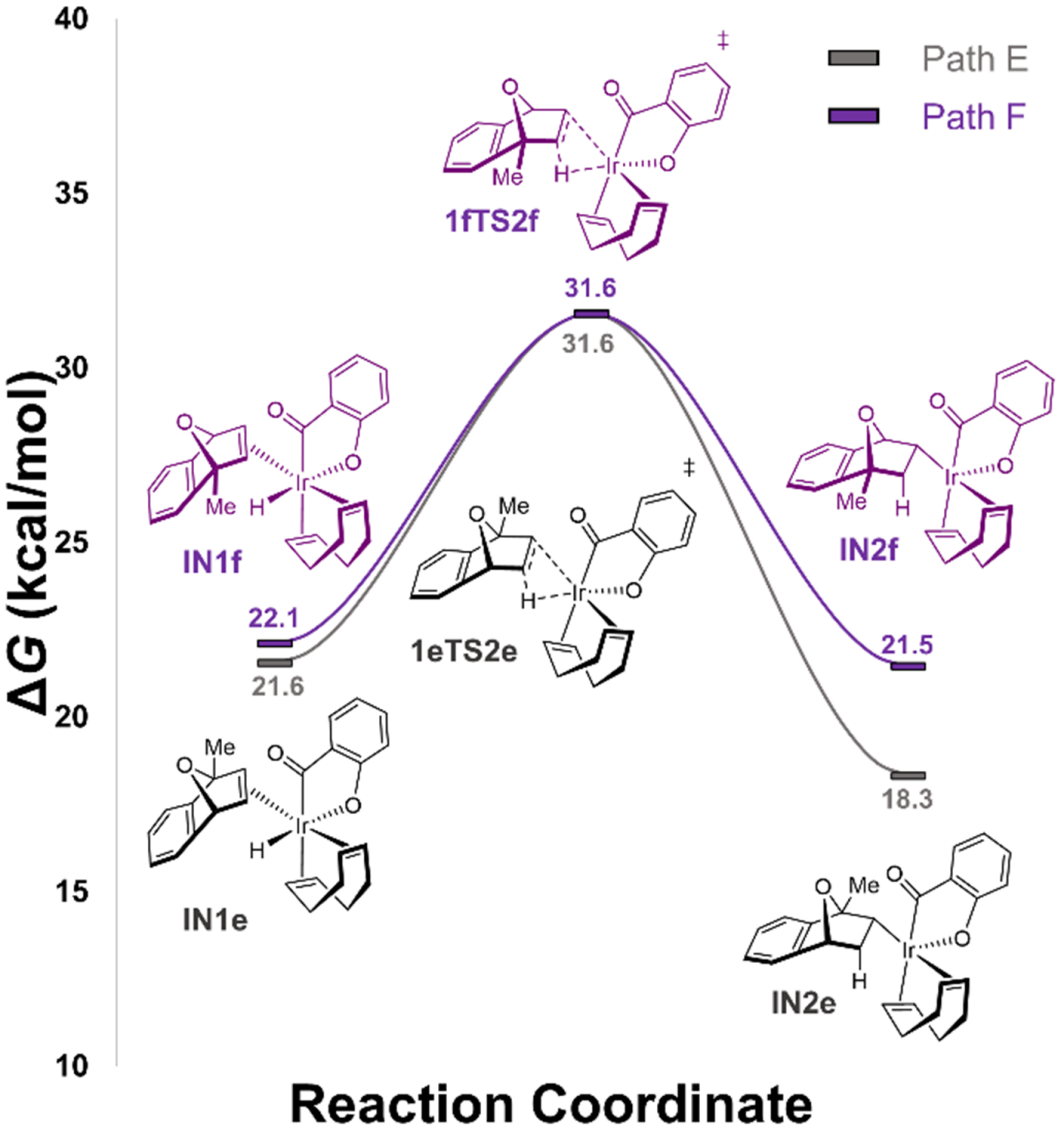
Potential energy profile of the PCM solvation model for the *endo* hydrometalation/reductive elimination pathway of the Ir/diene-catalyzed hydroacylation of MeOBD with salicylaldehyde (**14**) in 1,4-dioxane, as evaluated by DFT calculation (M06 [69]/def2SVP [70]/PCM [72]//M06 [69]/def2TZVP [70]/PCM [72]). Calculated Gibbs free energies (in kcal/mol; *T* = 338.15 K) are with respect to separated reactants Ir(COD)OH (Cat) + SaliK + MeOBD.

As mentioned above, it is typically assumed *exo*-η^2^-coordination is preferential over *endo*-η^2^-coordination; however, for greater completeness, we investigated the *endo*-hydroacylation of MeOBD as a potential competing reaction (Figure 3). There are two potential isomeric intermediates following *endo*-η^2^-coordination of MeOBD concerning the relative orientation of the COD ligand, acyl group, and C_1_-substituent on the oxabicyclic alkene. In **IN1e**, the chelated acyl group is positioned *syn* to the C_1_-methyl substituent while in **IN1f**, they are positioned *anti* to one another. Compared to the *exo*-η^2^-coordinated intermediates, the *endo*-isomers are 5.0 to 7.3 kcal/mol higher in energy. The concerning free energy barrier about **IN1e** to **IN2e**, via **1eTS2e** is 10.0 kcal/mol whereas it is 9.5 kcal/mol to form **IN1f** via **1fTS2f**. Although the calculated barriers are not prohibitively high, they are much greater than the *exo*-hydrometalation pathway. These results suggest the reaction’s stereoselectivity originates from the imposed steric constraints of the more-hindered *endo*-face.

**Figure 4:**
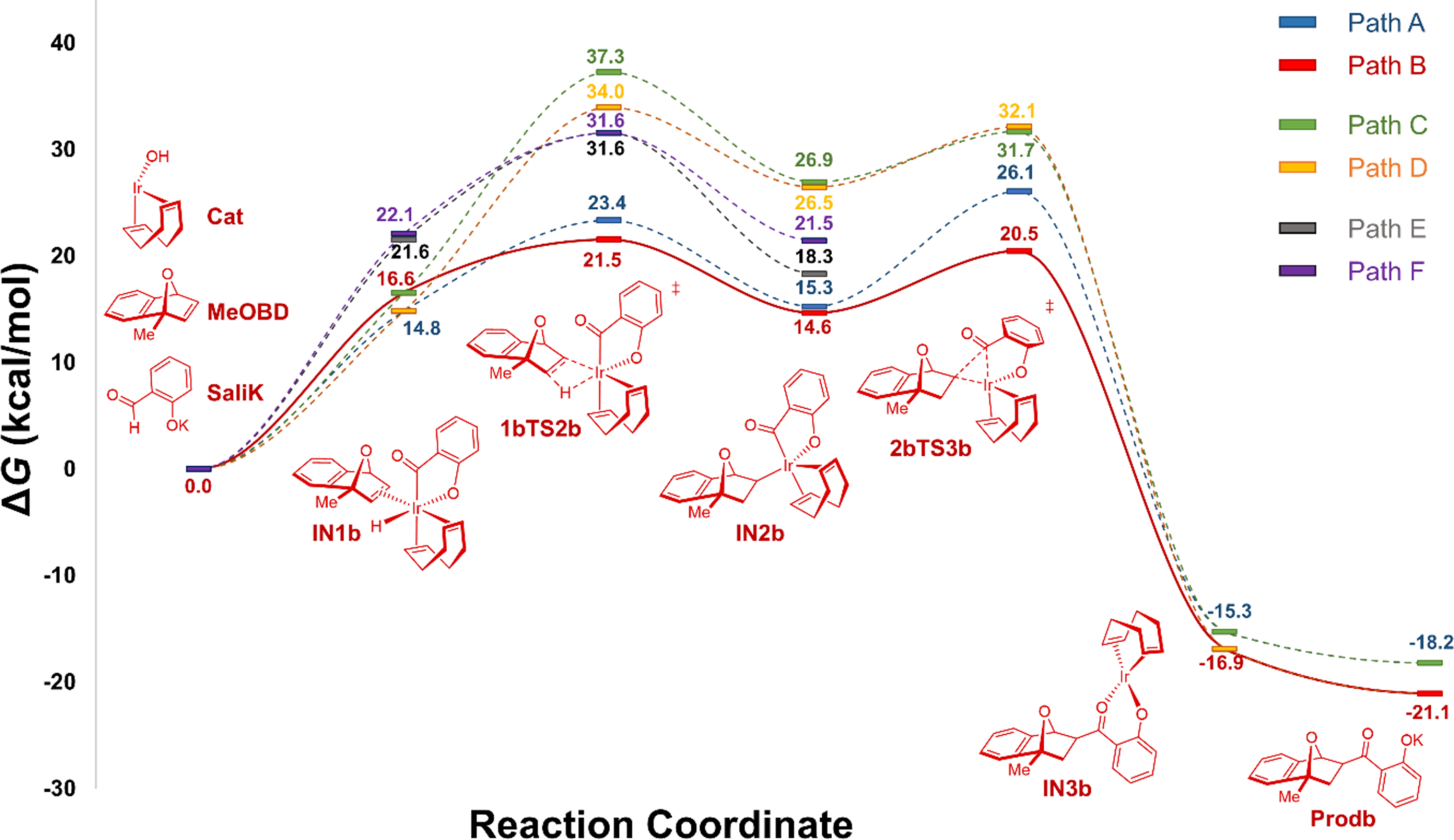
Potential energy profile of the PCM solvation model for the Ir/diene-catalyzed hydroacylation of MeOBD with salicylaldehyde (**14**) in 1,4-dioxane, as evaluated by DFT calculation (M06 [69]/def2SVP [70]/PCM [72]//M06 [69]/def2TZVP [70]/PCM [72]). Calculated Gibbs free energies (in kcal/mol; *T* = 338.15 K) are with respect to separated reactants Ir(COD)OH (cat) + SaliK + MeOBD.

Based on the calculated energies of the optimized intermediates and transition states for the three investigated pathways, an overall reaction mechanism can be proposed. It is predicted pathway B is the most accessible pathway for the hydroacylation reaction which corresponds with the production of the experimentally observed C_3_-*exo*-product **15**. First, the active iridium catalyst will undergo oxidative addition into the aldehyde C–H bond (Figure 4). Next, the iridium hydride species will undergo *exo*-η^2^-coordination with the olefin of MeOBD to generate intermediate **IN1b**. Insertion of the olefin into the iridium-hydride bond to form hydrometalated intermediate **IN2b** proceeds via **1bTS2b** which exhibits a distorted Ir–H–C–C’ four-membered ring geometry, requiring an activation of 4.9 kcal/mol. Reductive elimination through **2bTS3b** crosses an energy barrier of 5.9 kcal/mol to generate the extremely stable Ir(I) alkoxide intermediate **IN3b**. The final reductive elimination step possesses the greatest energy of activation in pathway B, acting as both the RDS and the origin of regioselectivity.

## Conclusion

In conclusion, we have successfully investigated the regioselectivity of iridium-catalyzed hydroacylation reactions of C_1_-substituted OBDs with salicylaldehyde. Utilizing commercially available [Ir(COD)Cl]_2_ in the presence of 5 M KOH in dioxane at 65 °C, a variety of hydroacylated bicyclic adducts were obtained in up to a 95% yield with complete stereo- and regioselectivity. It was observed the addition of the acyl group occurred entirely at the less hindered position, exclusively producing the C_3_-*exo*-product. Experimental and theoretical studies were undertaken in order to understand the mechanism. Although not fully discerned, electron-withdrawing C_1_-substituents seem to deactivate the catalyst, leading to severely diminished product yields. Using DFT calculations, we investigated the [Ir(COD)OH]_2_-catalyzed hydroacylation reactions of C_1_-substituted OBDs. From these results, we found the reductive elimination step is the rate-determining step and the origin of regioselectivity for the catalytic cycle. Moreover, we determined the stereoselectivity of the reaction arises from the unattractive interactions imposed from the sterically hindered *endo*-face of the bicyclic alkene. The mechanistic insights gained from this combined experimental and theoretical study will facilitate further future methodology development in this field.

## Supporting Information

File 1 Experimental procedures, compound characterization, and ^1^H and ^13^C NMR spectra of compounds.

File 2 Cartesian coordinates and selected energy values for all calculated structures.
