# Supplementary material for: Iridium-catalyzed hydroacylation reactions of C1-substituted oxabenzonorbornadienes with salicylaldehyde: an experimental and computational study
Source: Beilstein J Org Chem. 2022 Mar 2;18:251–61. doi: 10.3762/bjoc.18.30 (PMC8919423; doi:10.3762/bjoc.18.30)
Supplement: File 1 — Experimental procedures, compound characterization, and 1H and 13C NMR spectra of compounds. [file Beilstein_J_Org_Chem-18-251-s001.pdf]

BEILSTEIN JOURNAL OF ORGANIC CHEMISTRY

Supporting Information

for

Iridium-catalyzed hydroacylation reactions of C1-substituted oxabenzonorbornadienes with salicylaldehyde: an experimental and computational study

Angel Ho, Austin Pounder, Krish Valluru, Leanne D. Chen and William Tam

Beilstein J. Org. Chem. **2022,** *18,* 251–261. doi:10.3762/bjoc.18.30

Experimental procedures, compound characterization, and ^{1}H and ^{13}C NMR spectra of compounds

License and Terms: This is a supporting information file under the terms of the Creative Commons Attribution License (https://creativecommons.org/licenses/by/4.0). Please note that the reuse, redistribution and reproduction in particular requires that the author(s) and source are credited and that individual graphics may be subject to special legal provisions.
The license is subject to the *Beilstein Journal of Organic Chemistry* terms and conditions: (https://www.beilstein-journals.org/bjoc/terms)

Table of contents

General considerations S1

General procedure for the iridium-catalyzed hydroacylation reaction of C_1-substituted oxabenzonorbornadienes..........S2

Characterization data for compounds **15c**–**k** S2

References S5

^{1}H and ^{13}C NMR spectra for new compounds **15c**–**k**..........S6

General considerations

All hydroacylation reactions were carried out in screw-capped vials and did not need to be under inert atmosphere. All glassware was oven dried overnight before use. Commercial reagents were all used as received from their respective suppliers. Flash column chromatography was performed on 230–400 mesh silica gel purchased from Silicycle. Analytical TLC was performed on pre-coated silica gel 250 μm 60 F254 aluminum plates purchased from Silicycle. TLC visualization was carried out under UV light and *p*-anisaldehyde stain. Infrared samples were acquired as solids or as neat oils on a Bruker ALPHA platinum single reflection diamond ATR spectrophotometer and are reported in wave numbers (cm^{-1}). ^{1}H and ^{13}C NMR spectra were recorded on a Bruker Advance 400 MHz spectrometer ($CDCl_3$: δ 7.24 ppm (^{1}H at 400 MHz) or δ 77.0 ppm (^{13}C at 100 MHz)). HRMS analyses were performed at the Queen's Mass Spectrometry and Proteomics Unit, Kingston, Ontario. The samples were ionized by electron ionization (EI) or positive electrospray ionization (ESI). The synthesis of the bicyclic starting materials followed established literature procedures [1-3]. The characterization of **15a** and **15b** were previously reported by Nishimura and coworkers [4].

General procedure for the iridium-catalyzed hydroacylation reaction of C_1-substituted oxabenzonorbornadienes.

In a small, dried screw-cap vial containing a stirring bar, $[Ir(CODCl]_2$ was added (1.2 equiv). C_1-substituted OBD (1.0 equiv.) was dissolved in dioxane (1 mL) and added to the reaction mixture. Salicylaldehyde (27 μL, 1 equiv) was also dissolved in dioxane (1 mL) and added to the reaction mixture followed by the direct addition of 5 M KOH (10 mol %) The vial was sealed and secured tightly with polytetrafluoroethylene (PTFE) thread-seal tape. The reaction mixture was heated to 65 °C with continuous stirring for 20 h. The crude product was directly loaded onto a chromatography column and purified (EtOAC/hexanes).

Characterization data for compounds **15c–k**

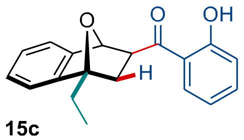

15c

Adduct 15c (Scheme 2): Yield: 52% (45.1 mg, 0.153 mmol); clear oil; R_f = 0.76 (EtOAc/hexanes 25:75)
IR (v, cm^{-1}): 3418,1639, 1486, 1446, 1348, 1275, 1156, 980, 894.
^{1}H NMR (CDCl$_3$, 400 MHz) δ: 12.33 (s, 1H), 7.63 (dd, 1H, J = 1.6, 7.9 Hz), 7.51-7.47 (m, 1H), 7.38-7.36 (m, 1H), 7.27-7.25 (m, 3H), 7.03 (dd, 1H, J = 0.9, 8.1 Hz), 6.91-6.87 (m, 1H), 5.66 (s, 1H), 3.54 (q, 1H, J= 4.3 Hz), 2.38-2.21 (m, 3H), 1.91 (dd, 1H, J= 8.9, 11.6 Hz), 1.17 (t, 3H, J= 7.2 Hz)
^{13}C NMR (100 MHz, CDCl$_3$) δ: 205.33, 163.15, 148.18, 145.76, 136.45, 129.81, 127.21, 126.91, 119.10, 119.02 (2C), 118.91, 118.76, 89.66, 79.93, 50.53, 37.04, 24.36, 9.04
HRMS (ESI) calcd. for $C_{19}H_{18}O_3$ $(M+Na)^+$: 294.1256; found 294.1263.

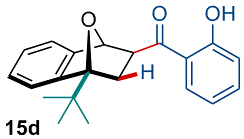

15d

Adduct 15d (Scheme 2): Yield: 76% (71.5 mg, 0.222 mmol); yellow solid; m.p. 114-126 °C; R_f= 0.47 (EtOAc/hexanes 25:75)
IR (v, cm^{-1}): 297, 1639, 1485, 1348, 1280, 1239, 1202, 1156, 1035, 1000, 943, 905
^{1}H NMR (CDCl$_3$, 400 MHz) δ: 12.34 (s, 1H), 7.68 (dd, 1H, J= 1.5, 8.1 Hz), 7.51-7.47 (m, 2H), 7.37-7.35 (m, 1H), 7.24-7.21 (m, 2H), 7.03 (dd, 1H, J= 1.2, 4.5 Hz), 6.93-6.89 (m, 1H), 5.58 (s, 1H), 3.51 (dd, 1H, J= 4.4, 8.7 Hz), 2.54 (dd, 1H, J= 4.4, 11.9 Hz), 1.78 (dd, 1H, J= 8.8, 11.2 Hz), 1.28 (s, 9H)
^{13}C NMR (100 MHz, CDCl$_3$) δ: 205.08, 162.14, 146.89, 146.29, 136.36, 129.75, 126.89, 126.61, 120.90, 119.09, 119.01, 118.78, 95.18, 79.91, 67.18, 50.62, 33.61, 32.51, 26.42 (3C)
HRMS (ESI) calcd. for $C_{21}H_{22}O_3$ $(M+Na)^+$: 322.1569; found 322.1573.

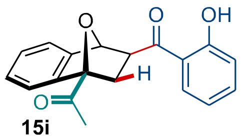

15i

Adduct 15i (Scheme 2): Yield: 5% (6.9 mg, 0.022 mmol); clear oil; R_f = 0.61 (EtOAc/hexanes 25:75)
IR (v, cm^{-1}): 2924, 1715, 1640, 1486, 1447, 1365, 1281, 1240, 1156, 1035, 933, 886

^{1}H NMR (CDCl$_3$, 400 MHz) δ: 12.18 (s, 1H), 7.59 (dd, 1H, J= 1.9, 8.1 Hz), 7.52-7.48 (m, 1H), 7.43-7.39 (m, 2H), 7.29-7.26 (m, 2H), 7.04 (d, 1H, J= 8.6 Hz), 6.91-6.87 (m, 1H), 5.79 (s, 1H), 3.58 (dd, 1H, J= 4.7, 8.5 Hz), 2.47 (dd, 1H, J= 4.9, 11.5 Hz), 2.41 (s, 3H)
^{13}C NMR (100 MHz, CDCl$_3$) δ: 206.29, 204.25, 163.22, 144.39, 144.07, 136.80, 129.71, 127.89, 127.69, 119.43, 119.34, 119.24, 119.07, 118.58, 91.85, 80.76, 49.43, 35.61, 26.97
HRMS (ESI) calcd. for $C_{19}H_{16}O_4$ $(M+Na)^+$: 308.1049; found 308.1039.

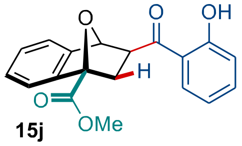

Adduct 15j (Scheme 2): Yield: 9% (11.8 mg, 0.036 mmol); clear oil; R_f = 0.78 (EtOAc/hexanes 25:75);
IR (ν, cm^{-1}): 2920, 1705, 1644, 1456, 1445, 1345, 1280, 1238, 1155, 1038, 950, 880
^{1}H NMR (CDCl$_3$, 400 MHz) δ: 12.17 (s, 1H), 7.51 (d, 1H, J= 7.9 Hz), 7.53-7.47 (m, 2H), 7.41-7.39 (m, 1H), 7.30-7.27 (m, 2H), 7.06-7.04 (m, 1H), 6.91-6.88 (m, 1H), 5.79 (s, 1H), 3.95 (s, 3H), 3.56 (dd, 1H, J= 4.9, 7.9 Hz), 2.69 (dd, 1H, J= 4.5, 11.3 Hz), 2.27 (dd, 1H, J= 8.6, 11.8 Hz)
^{13}C NMR (100 MHz, CDCl$_3$) δ: 203.58, 168.39, 163.24, 147.87, 147.37, 144.10, 143.76, 136.73, 129.59, 128.03, 127.74, 119.75, 119.29, 119.21, 119.18, 80.97, 67.22, 49.37, 36.22
HRMS (ESI) calcd. for $C_{19}H_{16}O_5$ $(M+Na)^+$: 324.0998; found 324.0986.

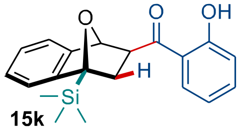

Adduct 15k (Scheme 2): Yield: 28% (29.3 mg, 0.087 mmol); clear oil; R_f = 0.51 (EtOAc/hexanes, 10:90)
IR (ν, cm^{-1}): 2957, 1639, 1486, 1447, 1347, 1250, 1201, 1156, 1034, 923, 842
^{1}H NMR (CDCl$_3$, 400 MHz) δ: 12.36 (s, 1H), 7.67 (dd, 1H, J= 1.7, 7.9 Hz), 7.50-7.46 (m, 1H), 7.38-7.36 (m, 1H), 7.27-7.25 (m, 1H), 7.21-7.19 (m, 2H), 7.03 (dd, 1H, J= 1.1, 8.7 Hz), 6.91-6.88 (m, 1H), 5.69 (s, 1H), 3.49 (dd, 1H, J= 4.0, 8.6 Hz), 2.39 (dd, 1H, J= 4.6, 11.7 Hz), 1.85 (dd, 1H, J= 8.9, 11.7 Hz), 0.29 (s, 9H)
^{13}C NMR (100 MHz, CDCl$_3$) δ: 205.49, 163.12, 149.19, 146.13, 136.36, 129.83, 127.05, 126.41, 119.47, 119.00 (2C), 118.92, 82.52, 81.73, 49.18, 35.19 (2C), 3.20 (3C)
HRMS (ESI) calcd. for $C_{20}H_{22}O_3Si$ $(M+Na)^+$: 338.1338; found 338.1325.

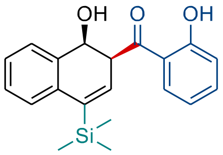

Adduct 16k (Scheme 2): Yield: 18% (18.2 mg, 0.054 mmol; clear oil; R_f = 0.43(EtOAc/hexanes 10:90)
IR (ν, cm^{-1}): 2957, 1638, 1486, 1447, 1348, 1250, 1205, 1159, 1035, 978, 921, 843
^{1}H NMR (CDCl$_3$, 400 MHz) δ: 11.76 (s, 1H), 8.03 (dd, 1H, J= 1.2, 8.2 Hz), 7.53-7.49 (m, 1H), 7.25 (d, 1H, J= 7.2 Hz), 7.19 (dt, 1H, J= 0.8, 7.2 Hz), 7.04-6.96 (m, 3H), 6.79 (d, 1H,

J= 7.2 Hz), 5.68 (d, 1H, J= 5.1 Hz), 4.31-4.29 (m, 1H), 2.22 (dd, 1H, J= 9.1, 11.7 Hz), 2.07 (dd, 1H, J= 3.8, 11.4 Hz), 0.31 (s, 9H)
^{13}C NMR (100 MHz, $CDCl_3$) δ:202.63, 162.65, 148.16, 142.16, 136.44, 129.97, 127049, 126.06, 120.31, 119.80, 119.30, 118.94, 118.76, 83.25, 83.19, 50.05, 32.21, 3.20 (3C)
HRMS (ESI) calcd. for $C_{20}H_{22}O_3Si$ $(M+Na)^+$: 339.1411; found 339.1404.

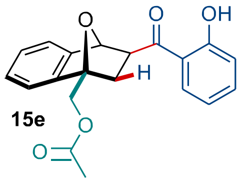
15e

Adduct 15e (Scheme 2): Yield: 95% (99.7 mg, 0.294 mmol); clear solid; m.p. 93-103°C; R_f = 0.75 (EtOAc/hexanes 25:75)
IR (v, cm^{-1}): 2950, 2360, 1739, 1641, 1447, 1348, 1229, 1037, 979, 884
^{1}H NMR ($CDCl_3$, 400 MHz) δ:12.23 (s, 1H), 7.61 (dd, 1H, J= 1.5, 7.9 Hz), 7.51-7.47 (m, 1H), 7.40-7.38 (m, 1H), 7.28-7.25 (m, 3H), 7.03 (dd, 1H, J= 0.9, 8.3 Hz), 6.92-6.87 (m, 1H), 5.71 (s, 1H), 4.95 (d, 1H, J= 12.5 Hz), 4.77 (d, 1H, J= 12.8 Hz), 3.56 (dd, 1H, J= 4.8, 8.8 Hz), 2.43 (dd, 1H, J= 4.5, 11.4 Hz), 2.11 (s, 3H), 1.92 (dd, 1H, J= 9.2, 11.1 Hz)
^{13}C NMR (100 MHz, $CDCl_3$) δ: 204.28, 170.87, 163.06, 144.92, 144.82, 136.52, 129.61, 127.46, 127.41, 119.24, 119.04, 118.97, 118.77, 87.17, 80.69, 49.06, 62.12, 49.81, 34.03, 20.77
HRMS (ESI) calcd. for $C_{20}H_{18}O_5$ $(M+Na)^+$: 338.1154; found 338.1159.

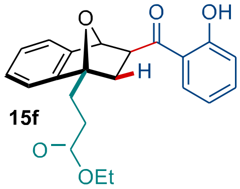
15f

Adduct 15f (Scheme 2): Yield: 52% (59.6 mg, 0.163 mmol); yellow oil; R_f = 0.44 (EtOAc/hexanes 23:75)
IR (v, cm^{-1}): 2981, 1730, 1641, 1446, 1182, 1095, 1034, 941, 895
^{1}H NMR ($CDCl_3$, 400 MHz) δ: 12.27 (s, 1H), 7.61 (dd, 1H, J= 1.6, 8.3 Hz), 7.50-7.46 (m, 1H), 7.37-7.35 (m, 1H), 7.27-7.25 (m, 3H), 7.03 (dd, 1H, J= 0.9, 8.3 Hz), 6.91-6.87 (m, 1H), 5.65 (s, 1H), 4.17-4.11 (m, 2H), 3.53 (dd, 1H, J= 4.7, 9.4 Hz), 2.74-4.52 (m, 1H), 2.27 (dd, 1H, J= 4.7, 11.6 Hz), 1.92 (dd, 1H, J= 9.1, 11.6 Hz), 1.24 (t, 3H, J= 7.1 Hz)
^{13}C NMR (100 MHz, $CDCl_3$) δ: 204.96, 173.43, 163.14, 146.45, 145.47, 136.50, 129.73, 127.36, 127.16, 119.16, 119.04, 119.03, 118.71, 118.65, 88.28, 80.06, 60.57, 50.52, 37.12, 29.72, 26.23, 14.28
HRMS (ESI) calcd. for $C_{22}H_{22}O_5$ $(M+Na)^+$: 366.1467; found 366.1473.

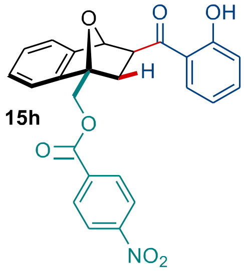
15h

Adduct 15h (Scheme 2): Yield: 82% (48.6 mg, 0.109 mmol); yellow solid; m.p. 180-188°C; R_f = 0.95 (EtOAc/hexanes 1:1)
IR (v, cm^{-1}): 3418, 1727, 1639, 1526, 1486, 1447, 1348, 1273, 1103, 1014, 872
^{1}H NMR ($CDCl_3$, 400 MHz) δ: 12.22 (s, 1H), 8.27-8.19 (m, 4H), 7.65 (dd, 1H, J= 1.5, 8.2 Hz), 7.52-7.48 (m, 1H), 7.44-7.42 (m, 1H), 7.33-7.27 (m, 3H), 7.04 (dd, 1H, J= 0.8, 8.5 Hz), 6.94-6.89 (m, 1H), 5.73 (s,

1H), 5.22 (d, 1H, J= 13.3 Hz), 5.11 (d, 1H, J= 11.9 Hz), 3.64 (dd, 1H, J= 4.6, 8.8 Hz), 2.59 (dd, 1H, J= 4.4, 11.3 Hz), 1.97 (dd, 1H, J= 8.8, 10.9 Hz)
^{13}C NMR (100 MHz, $CDCl_3$) δ: 203.99, 164.49, 163.14, 150.72, 144.91, 144.49, 136.62, 134.95, 130.99, 130.67, 129.50, 127.62, 127.53, 123.62, 123.55, 123.50, 119.39, 119.10, 118.62, 118.49, 87.26, 81.10, 63.33, 49.77, 33.79;
HRMS (ESI) calcd. for $C_{25}H_{19}O_7N$ $(M+Na)^+$: 446.1234; found 446.1238.

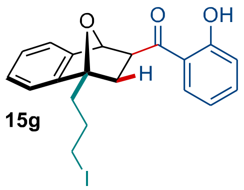

15g

Adduct 15g (Scheme 2): Yield: 54% (49.0 mg, 0.112 mmol); yellow oil; R_f = 0.61 (EtOAc/hexanes 25:75)
IR (v, cm^{-1}): 2947, 1638, 1579, 1486, 1446, 1349, 1274, 1156, 1013, 978, 941, 892
^{1}H NMR ($CDCl_3$, 400 MHz) δ: 12.37 (s, 1H), 7.60 (dd, 1H, J= 1.4, 8.0), 7.50-7.46 (m, 1H), 7.37-7.34 (m, 1H), 7.27-7.22 (m, 3H), 7.02 (dd, 1H, J= 1.2, 8.4 Hz), 6.90-6.86 (m, 1H), 5.64 (s, 1H), 3.52 (dd, 1H, J= 4.7, 8.8 Hz), 3.35-3.21 (m, 2H), 2.51-2.47 (m, 1H), 2.27-2.23 (m, 2H), 2.18-2.03 (m, 2H), 1.92 (dd, 1H, J= 8.6, 11.2 Hz)
^{13}C NMR (100 MHz, $CDCl_3$) δ: 205.06, 163.15, 146.72, 145.43, 136.54, 129.74, 147.36, 147.14, 119.17, 119.06, 118.82, 118.68, 88.52, 80.15, 67.19, 50.39, 39.51, 32.22, 28.74, 7.52
HRMS (ESI) calcd. for $C_{20}H_{19}O_3I$ $(M+Na)^+$: 435.0452; found 435.0449.

References

[1] Webster, R.; Böing, C.; Lautens, M. *J. Am. Chem. Soc.* **2009**, *131*, 444.
[2] Carlson, E.; Boutin, R.; Tam, W. *Tetrahedron* **2018**, *74*, 5510.
[3] Carlson, E.; Haner, J.; Mckee, M.; Tam, W. *Org. Lett.* **2014**, *16*, 1776.
[4] Nagamoto, M.; Mishimura, T. *Chem. Commun*. **2015**, *51*, 13791.

^{1}H and ^{13}C NMR spectra for new compounds **15c**–**k**

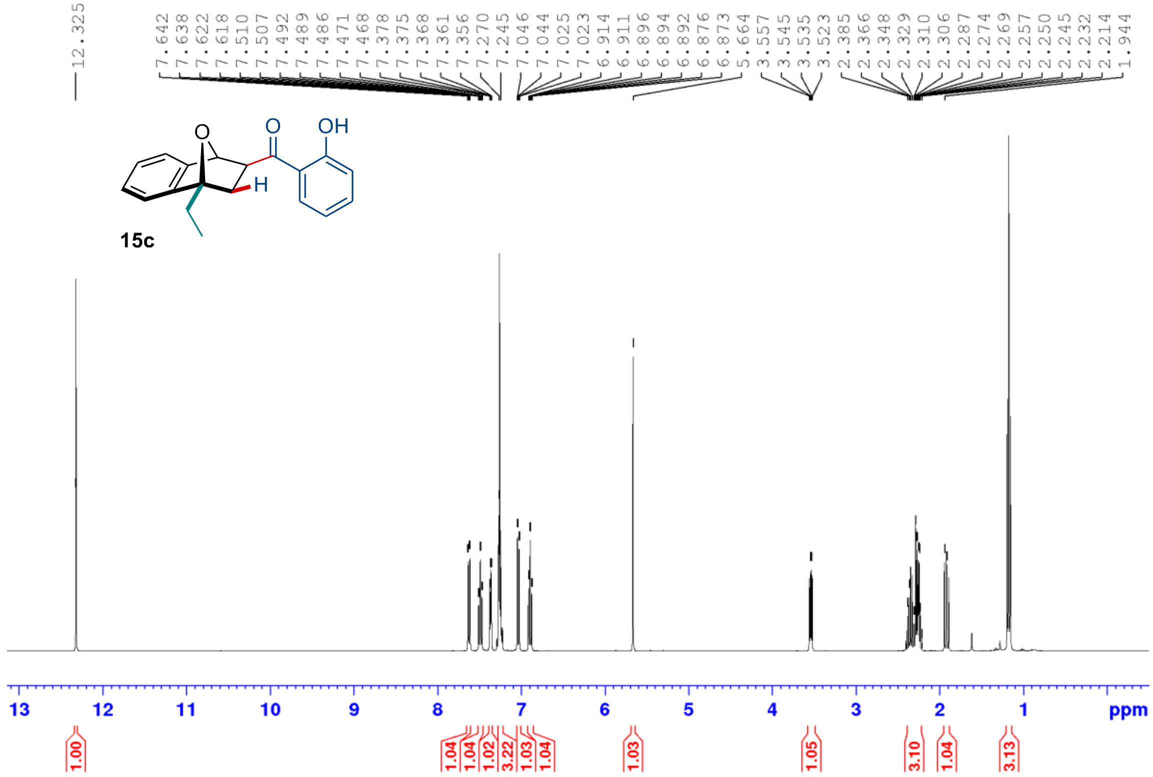

400 MHz ^{1}H NMR spectrum of **15c** in $CDCl_3$

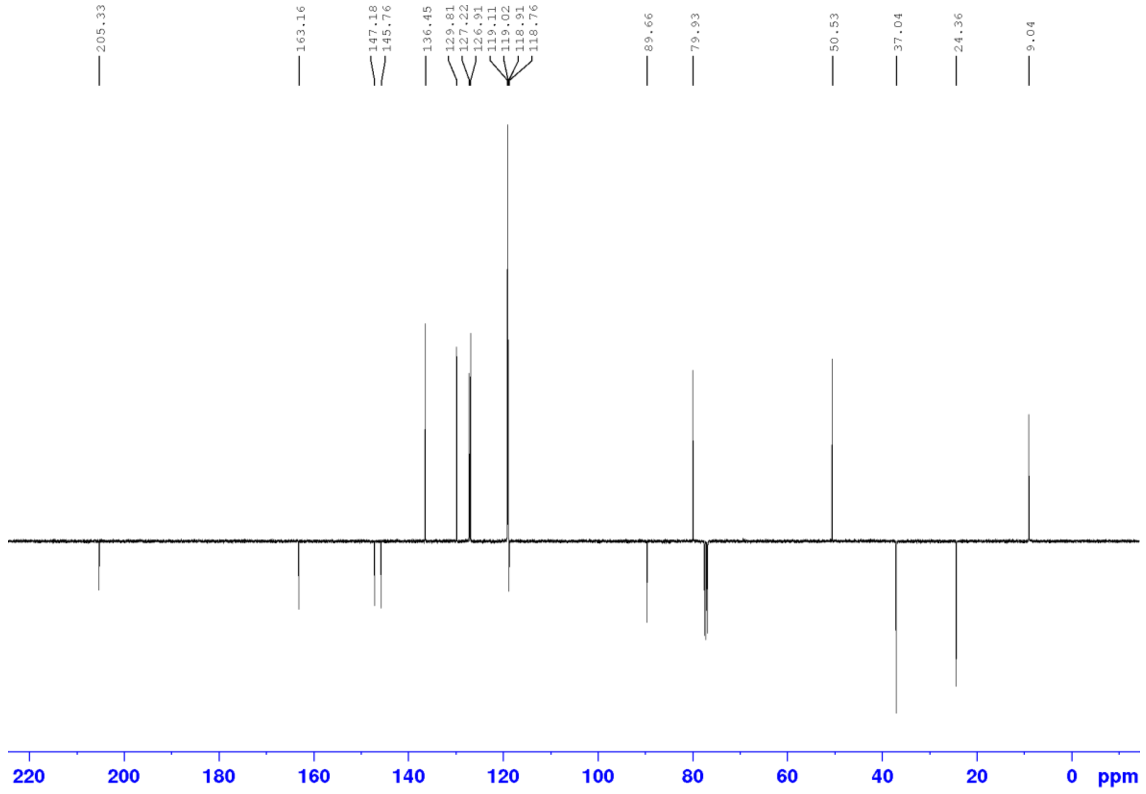

100 MHz ^{13}C NMR spectrum of **15c** in $CDCl_3$

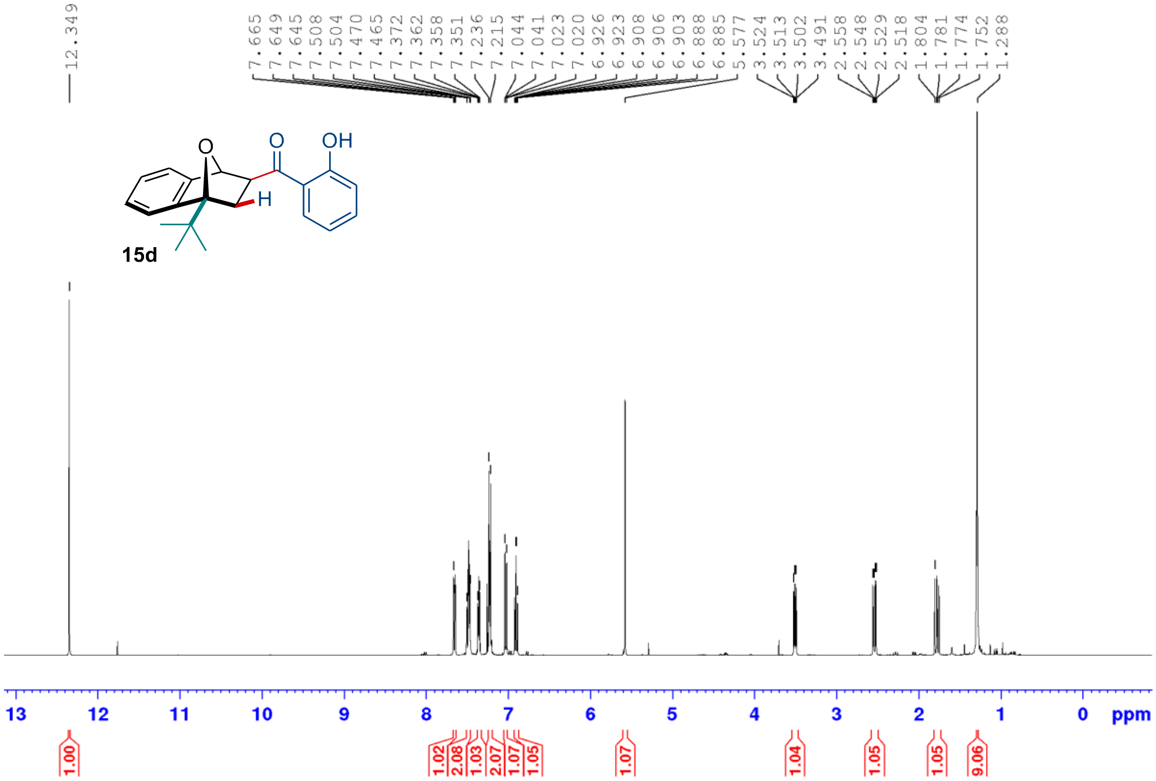

400 MHz ^{1}H NMR spectrum of **15d** in $CDCl_3$

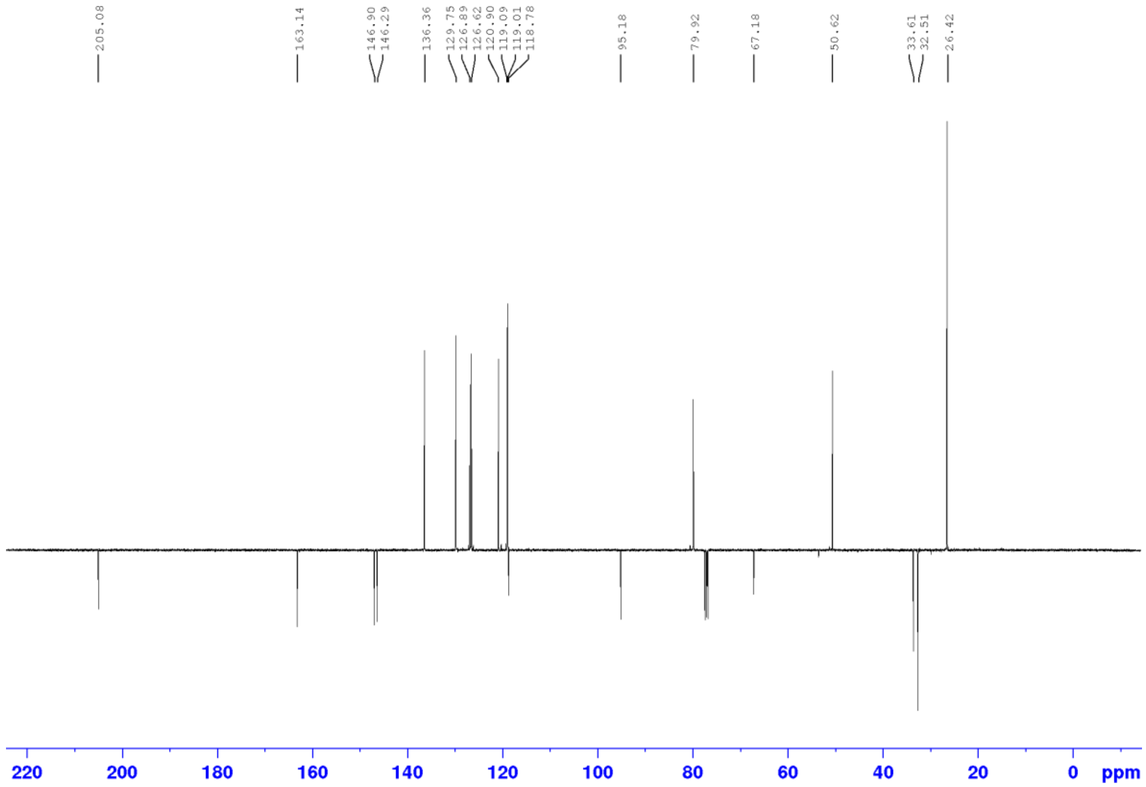

100 MHz ^{13}C NMR spectrum of **15d** in $CDCl_3$

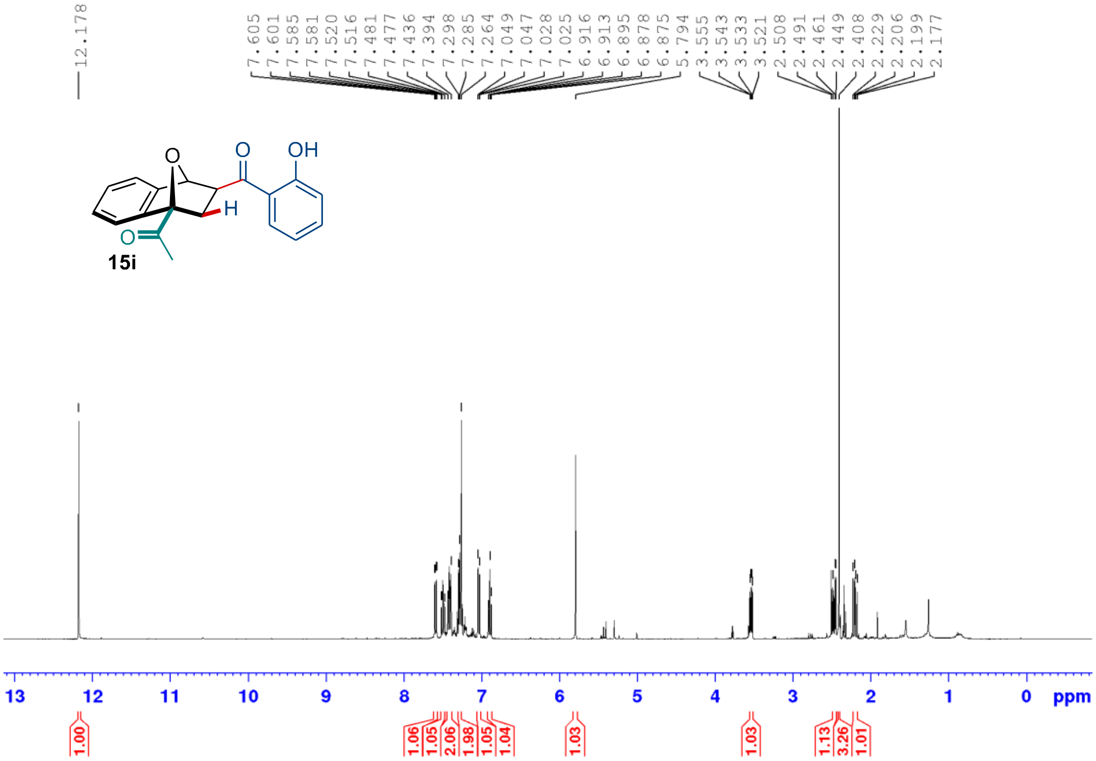

400 MHz ^{1}H NMR spectrum of **15i** in $CDCl_3$

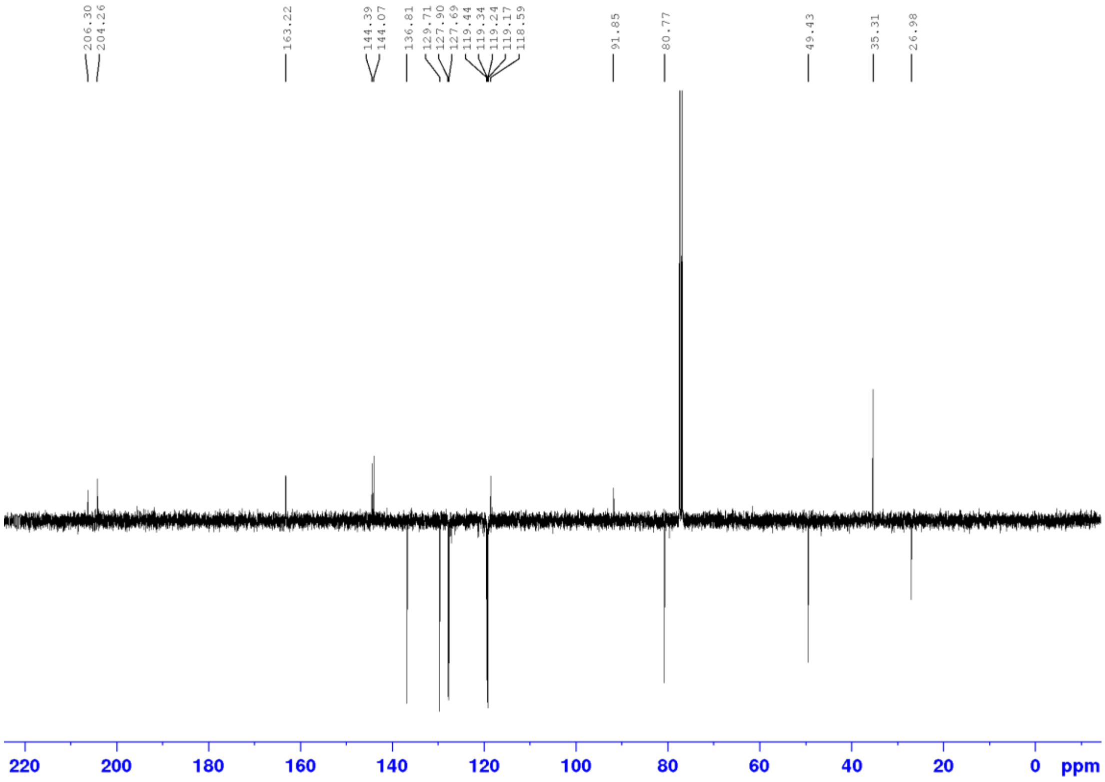

100 MHz ^{13}C NMR spectrum of **15i** in $CDCl_3$

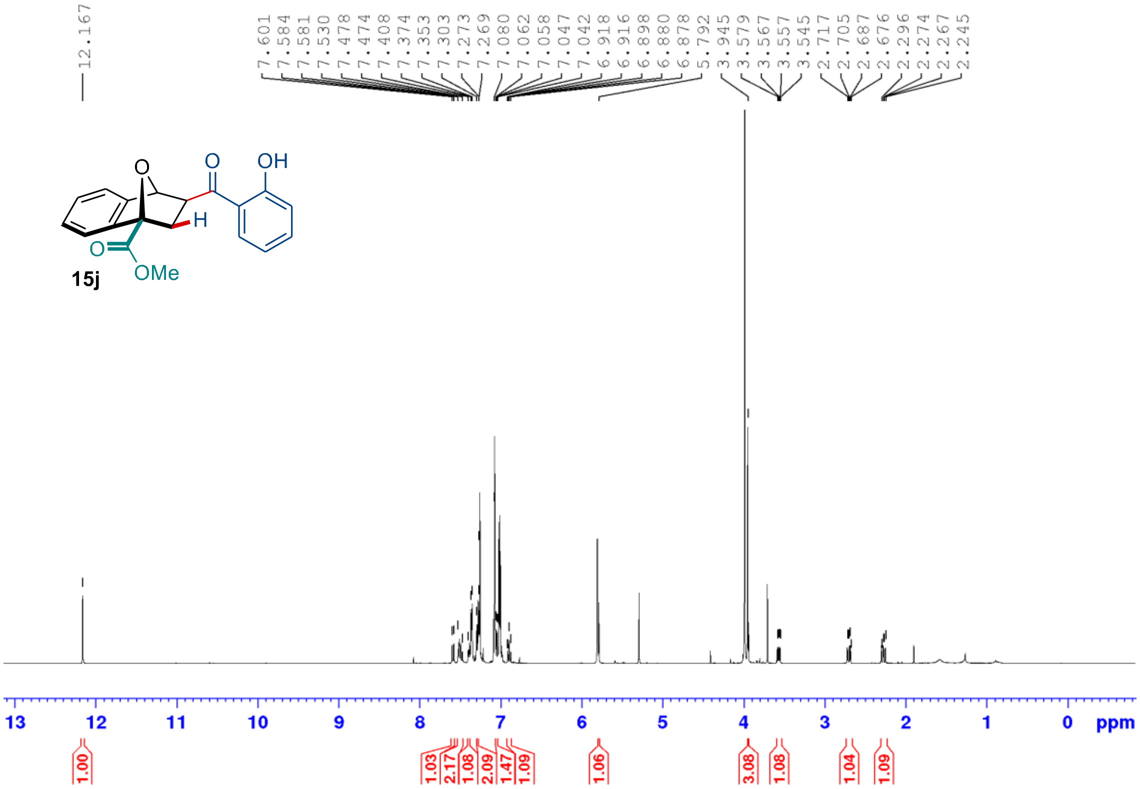

15j

400 MHz ^{1}H NMR spectrum of **15j** in $CDCl_3$

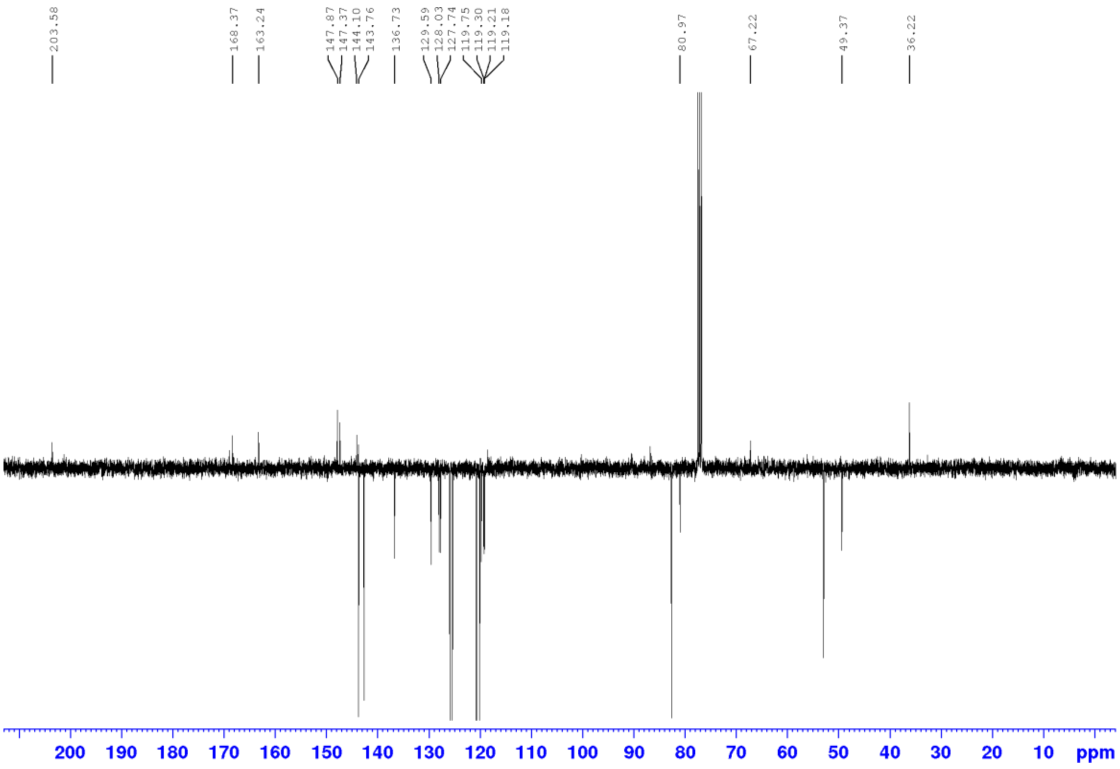

100 MHz ^{13}C NMR spectrum of **15j** in $CDCl_3$

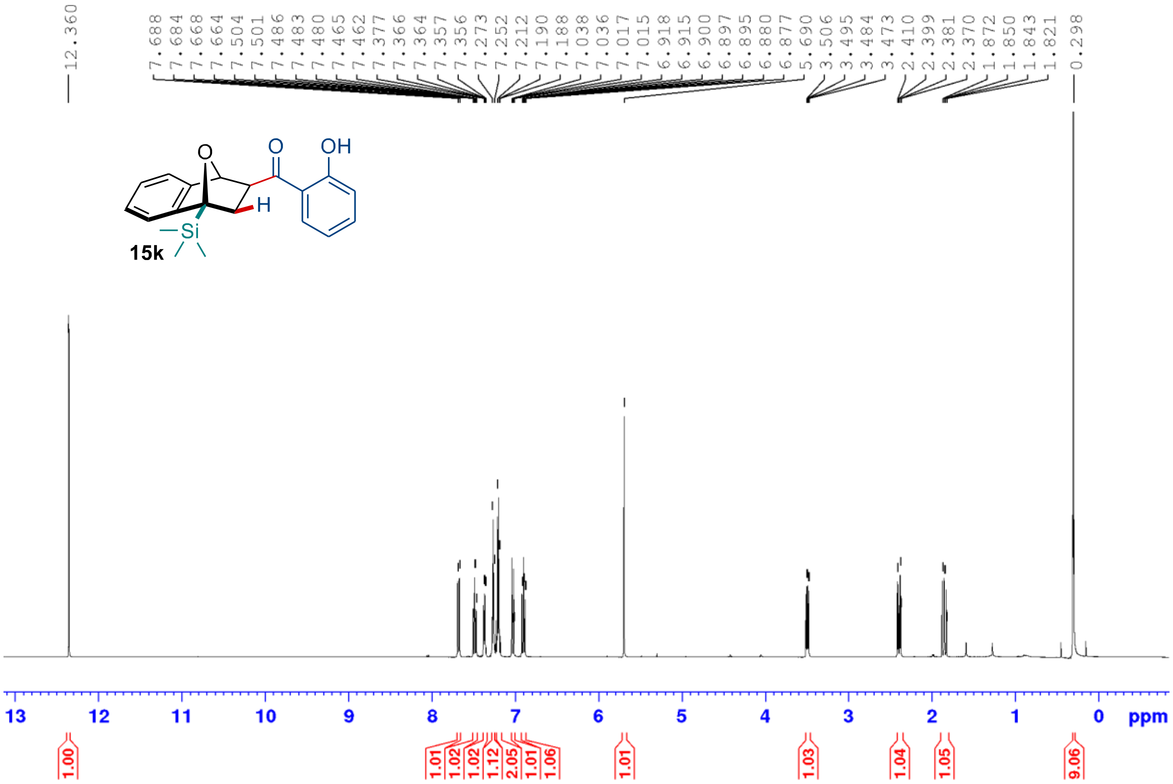

400 MHz ^{1}H NMR spectrum of **15k** in $CDCl_3$

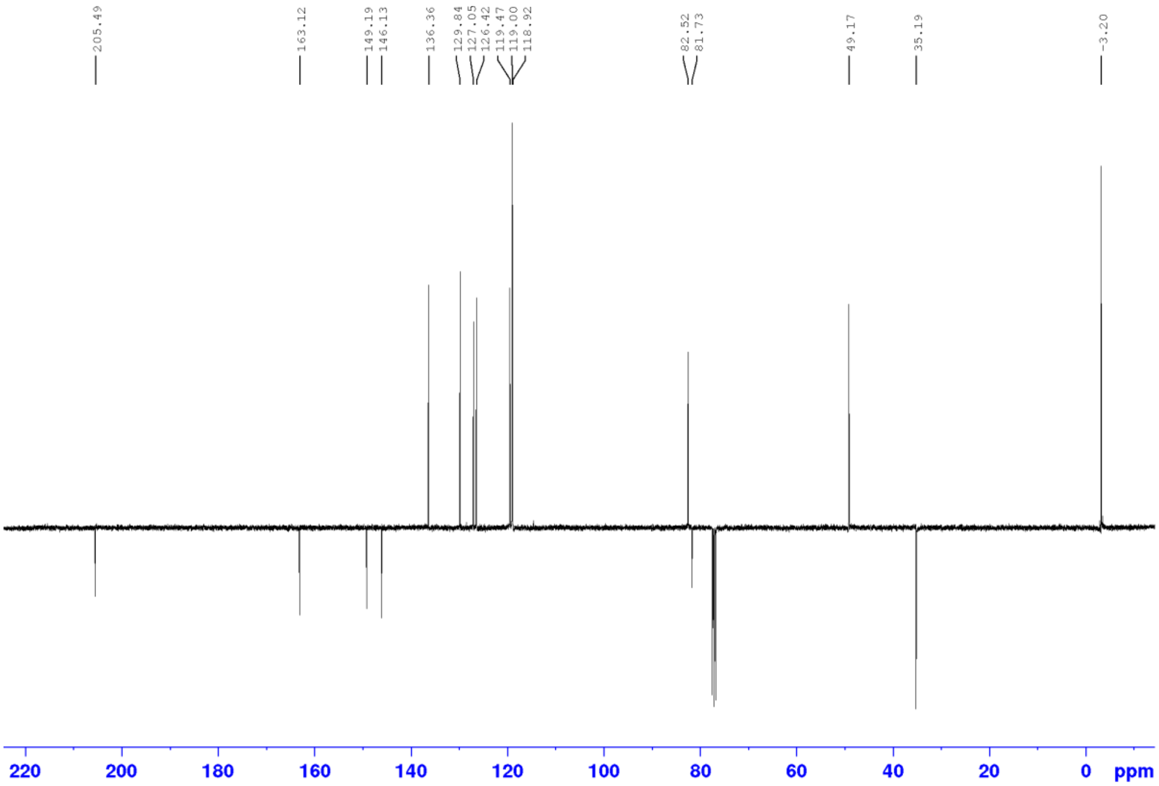

100 MHz ^{13}C NMR spectrum of **15k** in $CDCl_3$

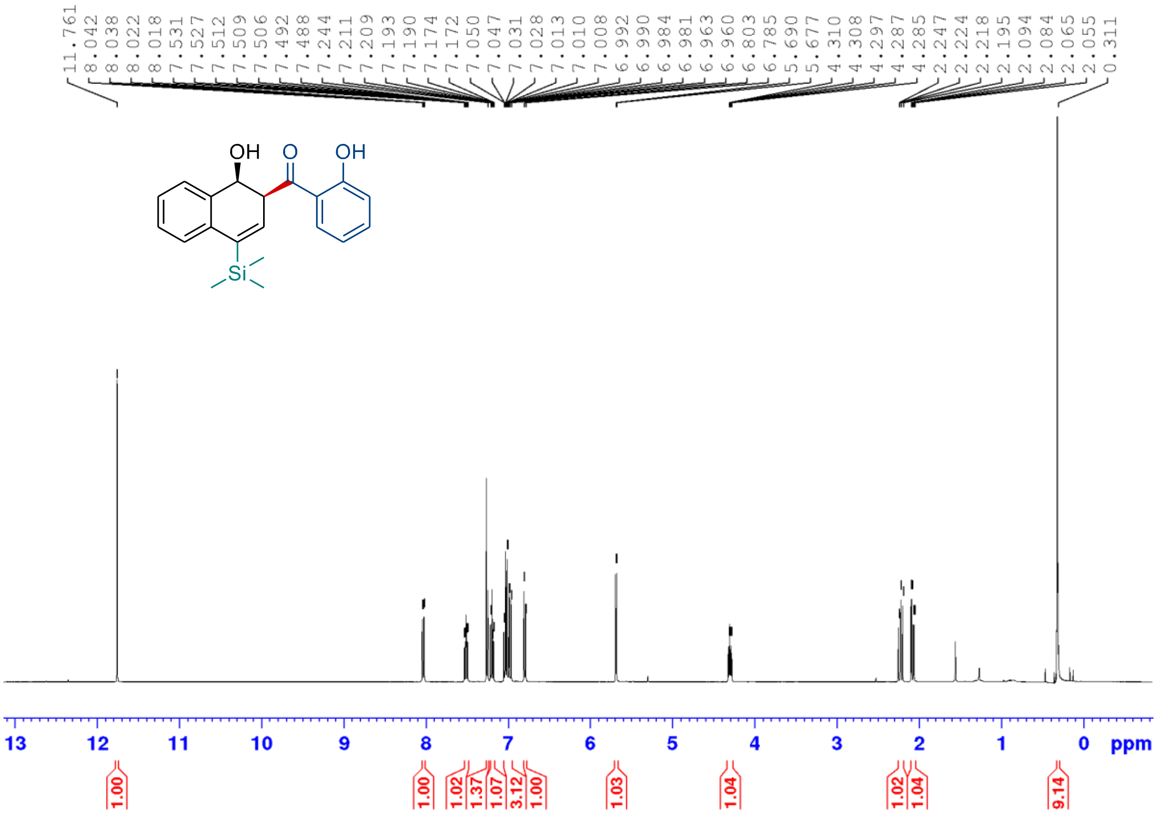

400 MHz ^{1}H NMR spectrum of **16k** in $CDCl_3$

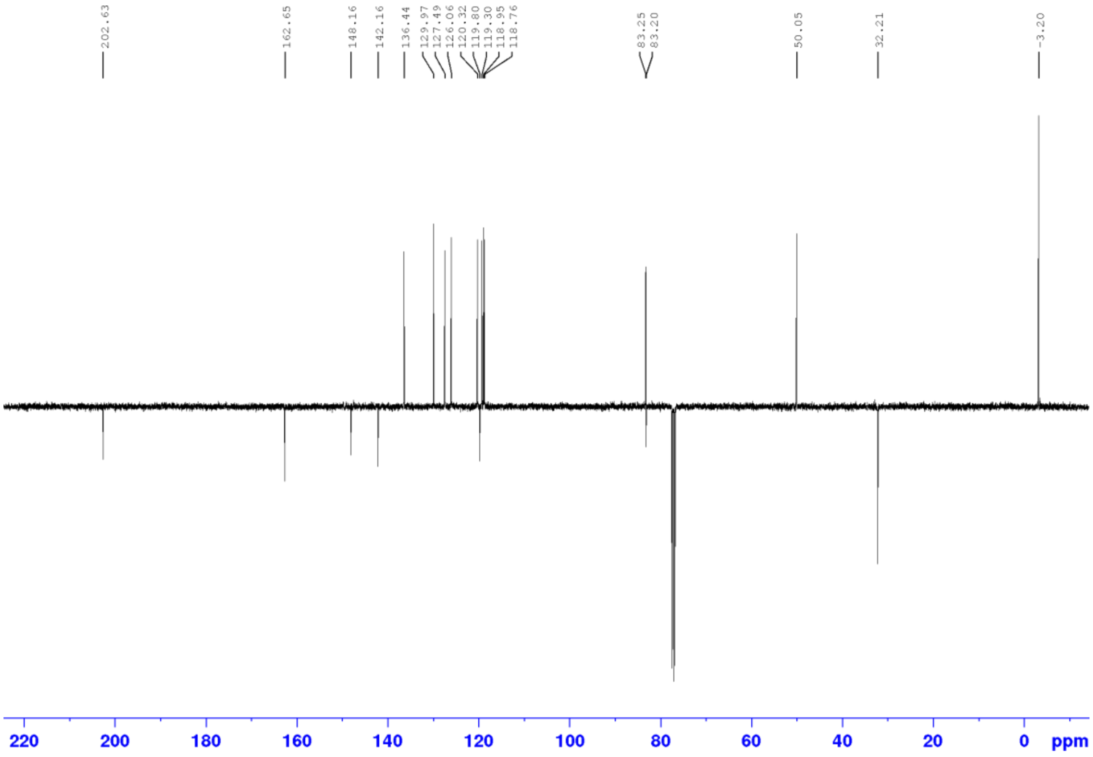

100 MHz ^{13}C NMR spectrum of **16k** in $CDCl_3$

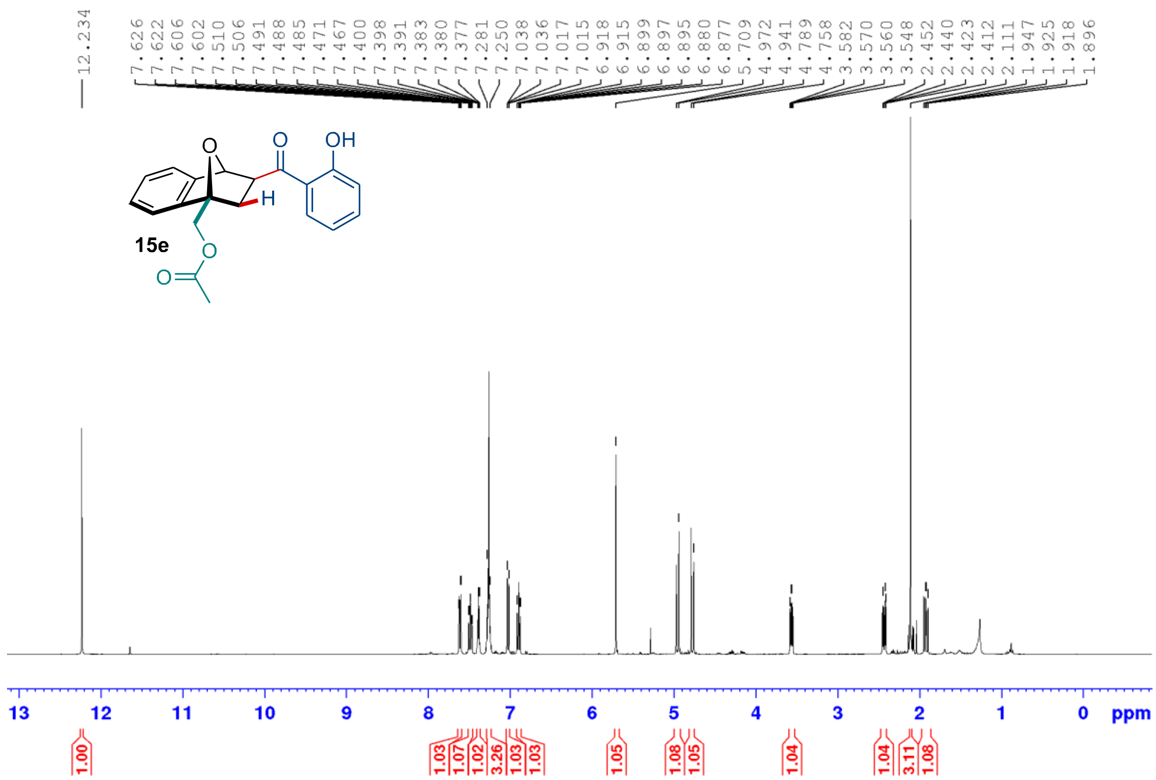

400 MHz ^{1}H NMR spectrum of **15e** in $CDCl_3$

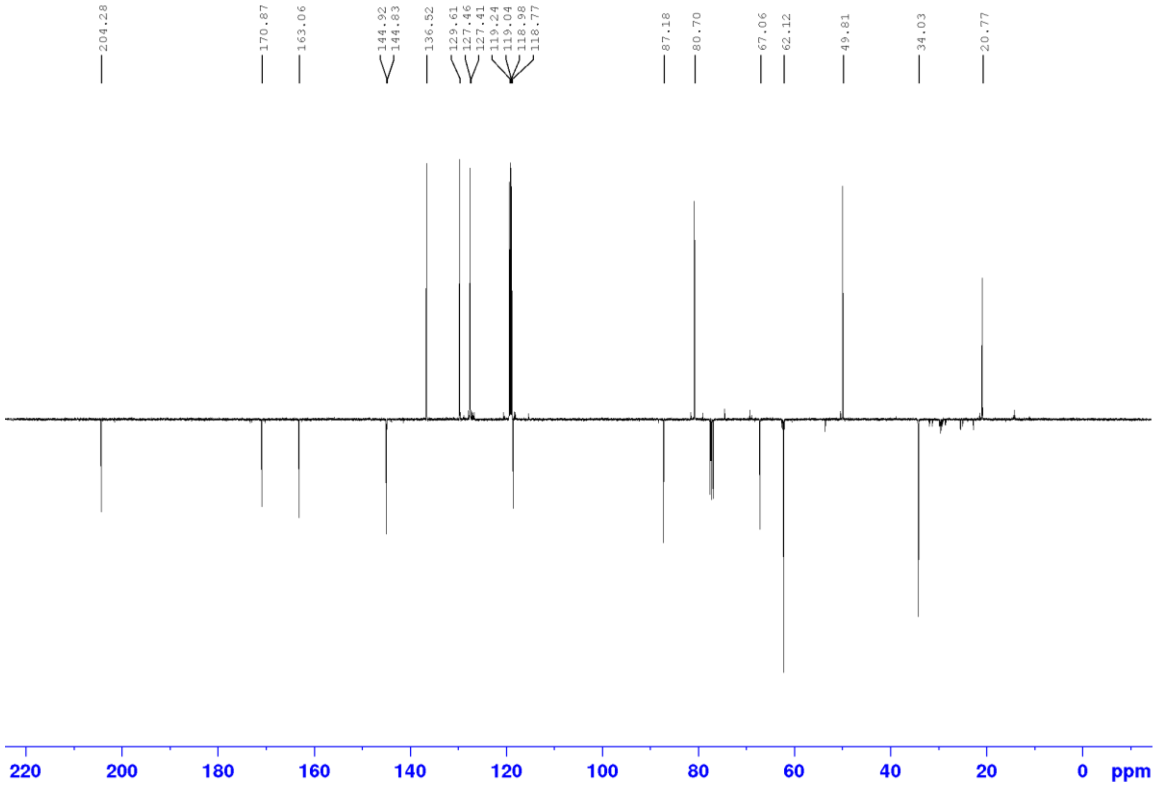

100 MHz ^{13}C NMR spectrum of **15e** in $CDCl_3$

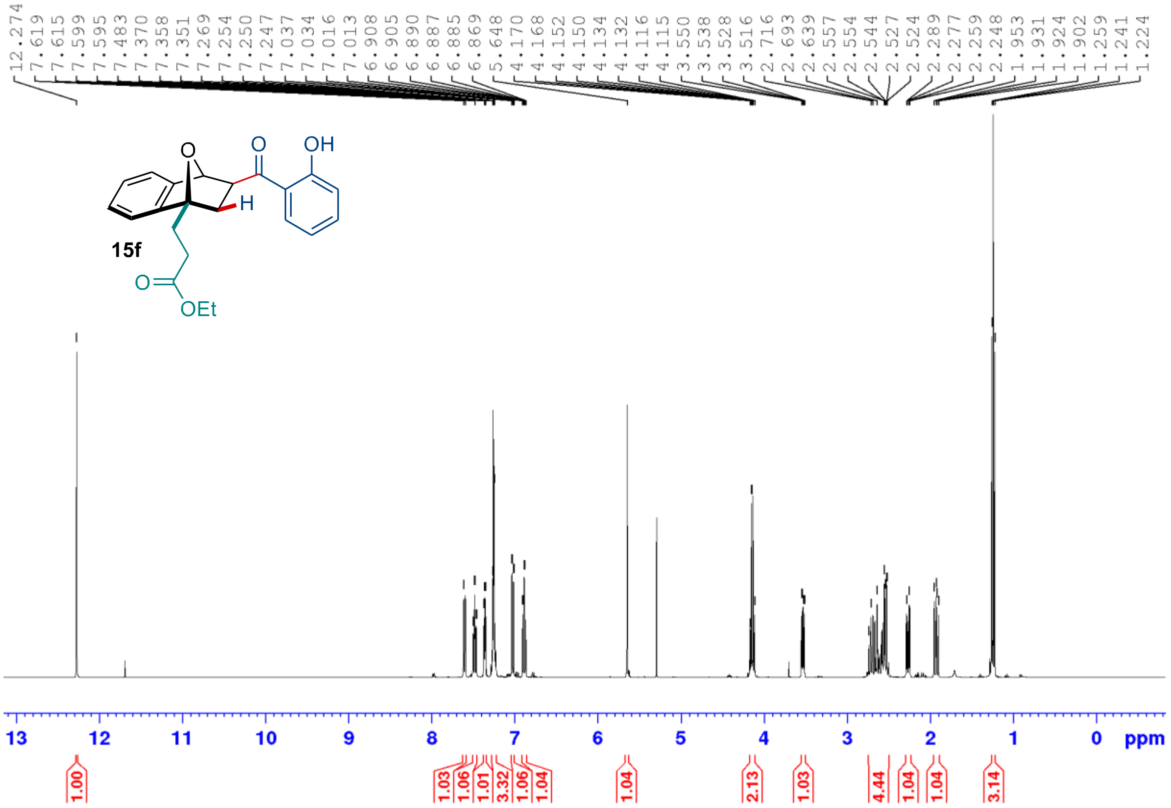

400 MHz ^{1}H NMR spectrum of **15f** in $CDCl_3$

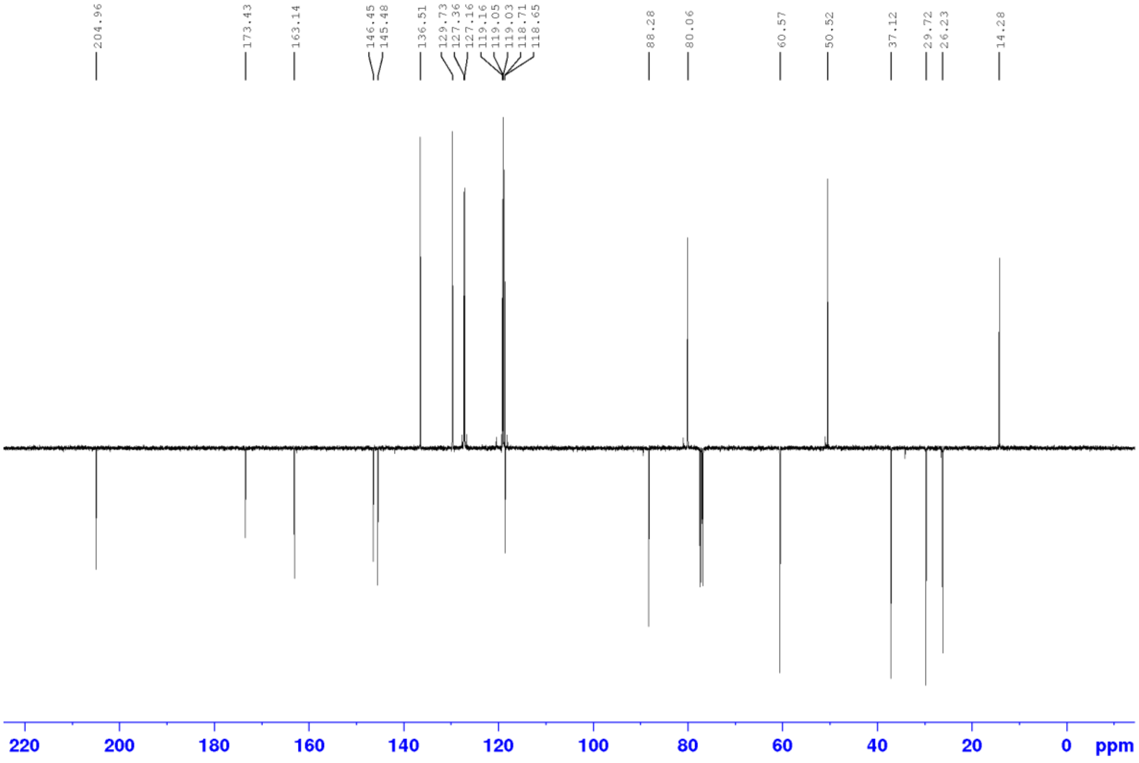

100 MHz ^{13}C NMR spectrum of **15f** in $CDCl_3$

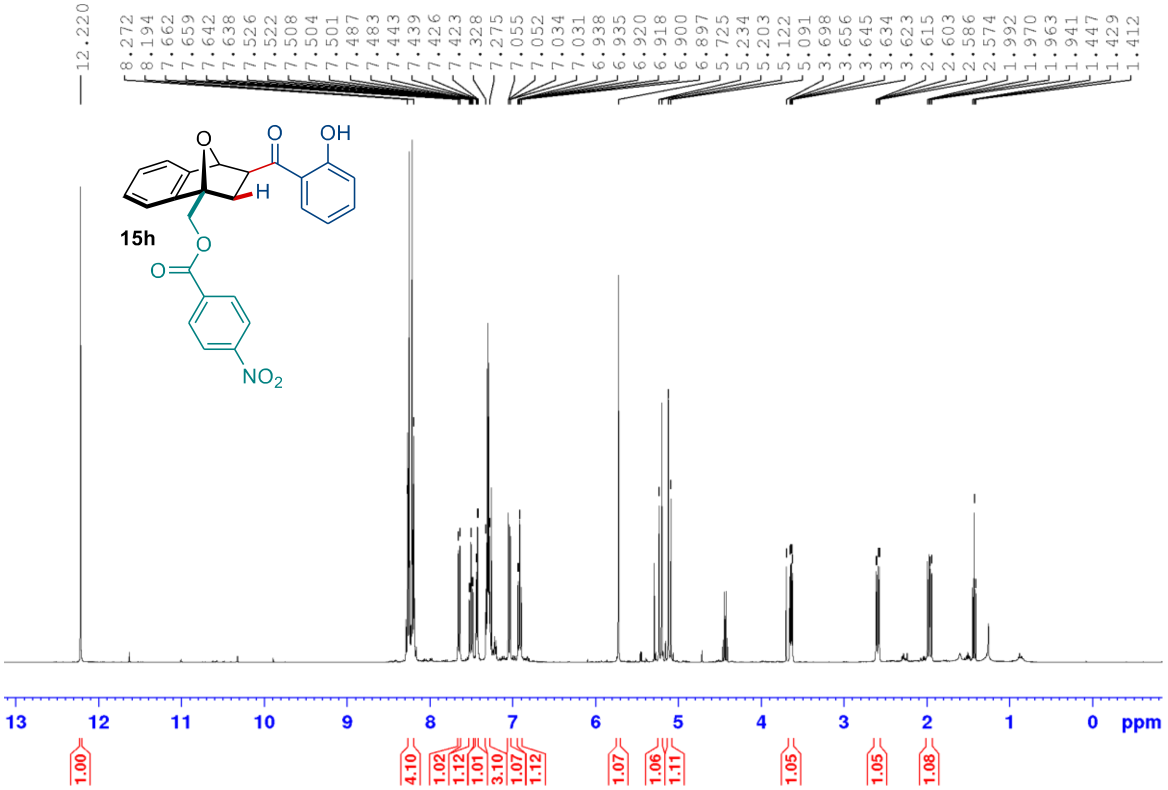

400 MHz ^{1}H NMR spectrum of **15h** in $CDCl_3$

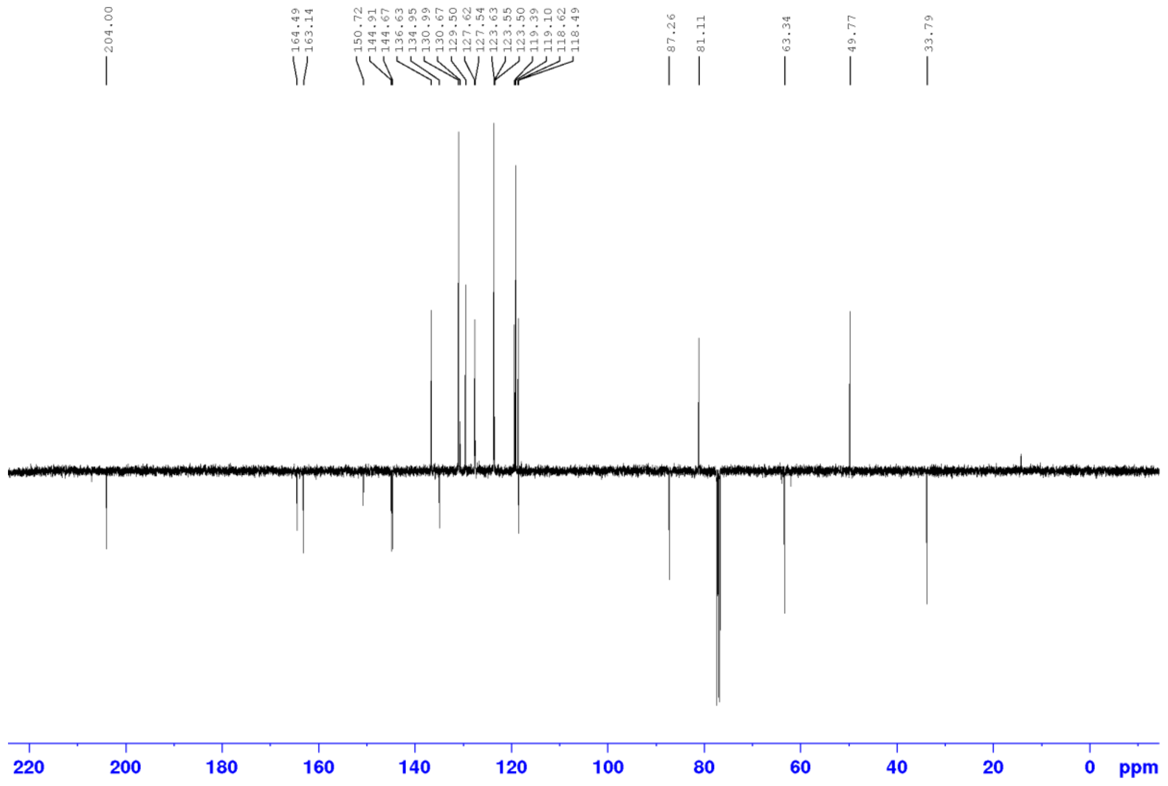

100 MHz ^{13}C NMR spectrum of **15h** in $CDCl_3$

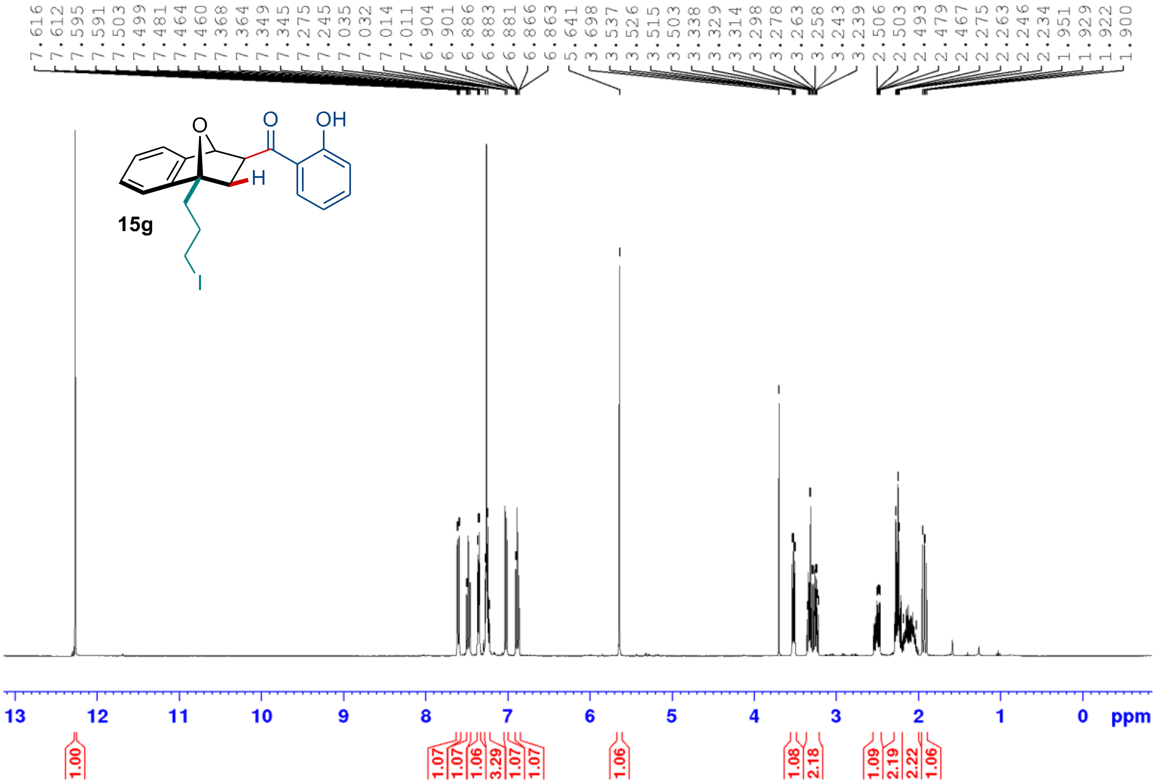

400 MHz ^{1}H NMR spectrum of **15g** in $CDCl_3$

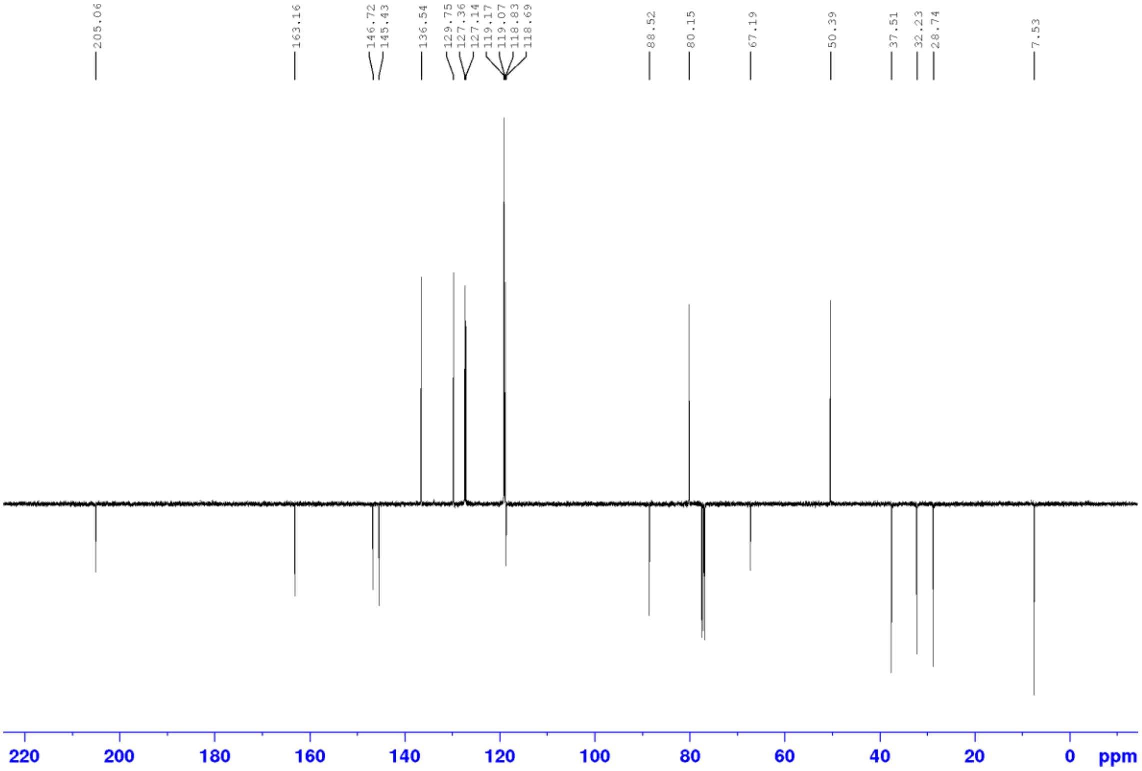

100 MHz ^{13}C NMR spectrum of **15g** in $CDCl_3$
