# Supplementary material for: Iridium-catalyzed hydroacylation reactions of C1-substituted oxabenzonorbornadienes with salicylaldehyde: an experimental and computational study
Source: Beilstein J Org Chem. 2022 Mar 2;18:251–61. doi: 10.3762/bjoc.18.30 (PMC8919423; doi:10.3762/bjoc.18.30)
Supplement: File 2 — Cartesian coordinates and selected energy values for all calculated structures. [file Beilstein_J_Org_Chem-18-251-s002.pdf]

BEILSTEIN JOURNAL OF ORGANIC CHEMISTRY

Supporting Information

for

Iridium-catalyzed hydroacylation reactions of C1-substituted oxabenzonorbornadienes with salicylaldehyde: an experimental and computational study

Angel Ho, Austin Pounder, Krish Valluru, Leanne D. Chen and William Tam

Beilstein J. Org. Chem. **2022,** *18,* 251–261. doi:10.3762/bjoc.18.30

Cartesian coordinates and selected energy values for all calculated structures

License and Terms: This is a supporting information file under the terms of the Creative Commons Attribution License (https://creativecommons.org/licenses/by/4.0). Please note that the reuse, redistribution and reproduction in particular requires that the author(s) and source are credited and that individual graphics may be subject to special legal provisions.
The license is subject to the *Beilstein Journal of Organic Chemistry* terms and conditions: (https://www.beilstein-journals.org/bjoc/terms)

Table of contents

Cartesian Coordinates and Selected Raw Output……………………………………………S2

Cartesian Coordinates and Selected Raw Output

Cat (Ir[COD]OH)$_2$

Electronic energy at 338.15 K (1,4-dioxane): -984.346550 au

Electronic energy + thermal free energy correction at 338.15 K (1,4-dioxane): -984.009733 au

```
H -1.49748900 1.02520700 2.26332400
C -2.19806000 0.97075500 1.41282600
C -2.46757200 2.31053300 0.76851900
C -3.07020700 -0.15532200 1.37639100
C -4.37660500 -0.61380200 -0.73209200
C -4.44028800 -0.12596300 0.71798700
H -2.95989400 -0.88305300 2.19474200
C -3.04020900 -0.32465200 -1.37417100
C -2.95258400 2.17299100 -0.67708200
C -2.37332600 0.93376600 -1.34002900
H -2.79575800 -0.98559500 -2.22119700
H -1.66537500 1.13633200 -2.16078400
H -1.51391500 2.86893000 0.77973100
H -3.17628300 2.90957100 1.37396600
H -2.66522800 3.06792100 -1.25233700
H -4.05507000 2.14018500 -0.72232700
H -5.20660400 -0.19125700 -1.33273500
H -4.51850400 -1.70834400 -0.75384200
H -5.13872800 -0.75603500 1.29201800
H -4.85952800 0.89408700 0.77271400
Ir -1.47408500 -0.39414700 0.01482400
O -0.01441300 -1.16662100 -1.29984300
H -0.02923800 -0.84847300 -2.21238800
H 1.49749700 1.02537700 -2.26338200
C 2.19785700 0.97085900 -1.41269900
C 2.46743100 2.31063300 -0.76847300
C 3.07012900 -0.15517800 -1.37634100
C 4.37675400 -0.61400300 0.73182600
C 4.44029600 -0.12583200 -0.71813200
H 2.95980000 -0.88273800 -2.19485000
C 3.04037500 -0.32479700 1.37393600
C 2.95256800 2.17308500 0.67712600
C 2.37358200 0.93370000 1.33999700
H 2.79596500 -0.98566400 2.22104000
H 1.66590000 1.13614300 2.16100900
H 1.51375500 2.86899600 -0.77947500
H 3.17601500 2.90967500 -1.37405300
H 2.66493200 3.06787700 1.25245500
H 4.05505700 2.14062200 0.72239100
H 5.20677400 -0.19165100 1.33256900
H 4.51855000 -1.70856600 0.75332000
H 5.13876900 -0.75560700 -1.29245200
H 4.85934900 0.89429600 -0.77262600
Ir 1.47405100 -0.39414000 -0.01481300
O 0.01459000 -1.16623000 1.30011400
H 0.02955400 -0.84791000 2.21257000
```

Half cat Ir[COD]OH

Electronic energy at 338.15 K (1,4-dioxane): --492.125033 au

Electronic energy + thermal free energy correction at 338.15 K (1,4-dioxane): -491.971714 au

```
H -0.68174700 -2.01751100 -1.55342800
C -0.99763000 -1.22268000 -0.85727000
C -1.79100900 -1.72109100 0.33181600
C -1.08698500 0.09133200 -1.39525000
C -1.34798100 2.03372000 0.18639600
C -2.04110900 1.13942900 -0.84540100
H -0.82214000 0.20619500 -2.45784900
C -0.25047600 1.30718900 0.92303600
C -1.68976100 -0.78017200 1.53730900
C -0.38623500 0.00230100 1.53062900
H 0.49762500 1.97551900 1.38008700
H 0.27608100 -0.19427500 2.38946200
H -1.38003900 -2.70716000 0.60985700
H -2.85062700 -1.90205700 0.06396900
H -1.76365200 -1.35900500 2.47168600
H -2.53812200 -0.07350300 1.56273400
H -2.07362800 2.46598400 0.90386600
H -0.88142400 2.89446500 -0.32339500
H -2.43425600 1.75784500 -1.66839900
H -2.92706700 0.64824300 -0.40560400
Ir 0.68599300 -0.11614300 -0.22154000
O 2.47100400 0.19955400 0.52455200
H 2.53663600 0.55163700 1.42155500
```

KOH

Electronic energy at 338.15 K (1,4-dioxane): -675.763153 au

Electronic energy + thermal free energy correction at 338.15 K (1,4-dioxane): -675.778517 au

```
O 0.00856800 1.48260400 0.00000000
H -0.23132900 2.40968100 0.00000000
K 0.00856800 -0.75108000 0.00000000
```

Salicylaldehyde-K

Electronic energy at 338.15 K (1,4-dioxane): -1020.067407 au

Electronic energy + thermal free energy correction at 338.15 K (1,4-dioxane): -1020.007902 au

```
C -1.76649800 0.74607800 0.00003700
C -2.20962600 -0.55475100 -0.00000100
C -1.30996000 -1.64990800 -0.00002400
C 0.04286200 -1.37370500 0.00000600
C 0.53919000 -0.05606500 -0.00000300
C -0.35505700 1.09759000 -0.00001000
H -2.47194100 1.58633300 0.00007400
H -3.29001400 -0.75239800 0.00000400
H -1.68277800 -2.67926700 -0.00002200
H 0.79118000 -2.17627900 -0.00001000
C 1.96753500 0.15570100 0.00002300
O 2.45629914 1.28068254 0.00002713
H 2.62963586 -0.74973354 0.00000287
O 0.03301300 2.28417900 -0.00003000
K 1.91914443 3.69439026 -0.33702233
```

MeOBD Coordinates

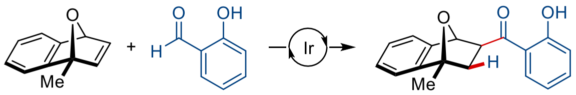

Main Path

MeOBD

Electronic energy at 338.15 K (1,4-dioxane): -500.168186 au

Electronic energy + thermal free energy correction at 338.15 K (1,4-dioxane): -500.029150 au

C 1.78331300 -0.21865000 1.15599200
C 1.28728400 0.56877600 -0.06955400
C -0.24358200 0.52852200 0.01322500
C -0.57026100 -0.81074300 -0.24724300
C 0.79064400 -1.47405000 -0.45980000
C 1.47888600 -1.49343400 0.91004800
H 2.21505600 0.22720200 2.05403000
H 1.59527000 -2.36726400 1.55227800
H 0.81305400 -2.39684500 -1.05471700
C 1.94091300 1.87730300 -0.39064000
H 1.76685400 2.60232600 0.41997200
H 3.02685000 1.74573700 -0.51127800
H 1.52956200 2.29202700 -1.32381300
C -1.88035700 -1.23873500 -0.25173500
C -2.88360500 -0.28560800 0.00562500
C -2.56003700 1.04123600 0.26136400
C -1.21879700 1.46796300 0.27078600
H -2.14393300 -2.28129600 -0.45551700
H -3.93344300 -0.59346700 -0.00074000
H -3.35788400 1.76476400 0.45356300
H -0.96870700 2.51558800 0.47003400
O 1.48886400 -0.41053200 -1.09902800

1a

Electronic energy at 338.15 K (1,4-dioxane): -1336.607747 au

Electronic energy + thermal free energy correction at 338.15 K (1,4-dioxane): -1336.206607 au

C 1.37175800 2.18255600 -0.97747400
C 1.70882300 3.29626800 -1.78997900
C 1.26640300 2.40358600 0.42305600
C 1.93175900 4.53597900 -1.21970800
H 1.78900100 3.14020500 -2.86998100
C 1.50099500 3.67159900 0.98094800
C 1.83283800 4.74156400 0.17002200
H 2.19333800 5.37948100 -1.86788000
H 1.41101100 3.77890100 2.06698700
H 2.01529400 5.73189700 0.59587700
C -1.25526700 -0.43816000 -0.88607900
C -2.31333600 -1.47834500 -0.48898200
C -3.64066200 -0.75085000 -0.67125600
C -3.66239100 0.16781500 0.38902400
C -2.34998100 -0.07578300 1.14072800
C -1.29976200 0.49400100 0.16509100
H -1.09424000 -0.15228300 -1.93027200
H -2.23355100 -2.49035700 -0.91067600
H -1.21724500 1.57736300 0.02758200
H 0.54306300 -1.66759100 -2.43530500
C 1.30655800 -1.93442100 -1.69504300
C 2.70678400 -1.52911000 -2.05485100
C 0.93787900 -2.78566200 -0.68304000
C 2.25743100 -2.79826700 1.49995100
C 1.90731300 -3.52681200 0.19997500
H -0.10664300 -3.11360800 -0.65008700
C 2.33121200 -1.30142500 1.38038200
C 3.61579900 -1.23104400 -0.85963800
C 2.92099700 -0.59117900 0.32671200
H 2.21886300 -0.76744600 2.33147500
H 3.23083500 0.43796200 0.54868500
O 1.16530500 1.00354400 -1.47623800
C 0.91300200 1.25536800 1.26861800
O 0.83688900 1.28394700 2.47556600
H 2.62665400 -0.62397300 -2.67561100
H 3.16279400 -2.30687800 -2.69757200
H 4.41923800 -0.55639800 -1.19377000
H 4.12954000 -2.14595200 -0.51709100
H 3.22096000 -3.17260800 1.89657000
H 1.50448800 -3.03517400 2.26959400
H 1.47641600 -4.50950900 0.44843200
H 2.82042400 -3.74400700 -0.37841800
Ir 0.69986000 -0.46083500 0.09962300
H 0.10038000 -1.33213600 1.27131000
C -2.27926700 0.29253700 2.58828500
H -1.30983400 -0.00263100 3.01514500
H -2.37302800 1.38403400 2.70497100
H -3.09390600 -0.19392200 3.14625000
C -5.74423400 0.07967000 -1.42603400
C -5.76667600 0.98975200 -0.37474400
C -4.71023900 1.05047800 0.55090000
C -4.66510800 -0.80545800 -1.59346700
H -6.57952600 0.05053400 -2.13178900
H -6.61901900 1.66658800 -0.26523000
H -4.72959900 1.77160200 1.37463400
H -4.65232900 -1.51664000 -2.42507100
O -2.21199500 -1.48637600 0.92566300

1b

Electronic energy at 338.15 K (1,4-dioxane): -1336.604994au

Electronic energy + thermal free energy correction at 338.15 K (1,4-dioxane): -1336.203866 au

C 1.53862000 2.15412800 -0.90084200
C 2.00855300 3.25050200 -1.66952900
C 1.36633900 2.36263200 0.49640200
C 2.29433700 4.46006900 -1.06304700
H 2.13867200 3.10565800 -2.74628000
C 1.66235600 3.60061400 1.09049900
C 2.12722700 4.65228300 0.32166800
H 2.66006700 5.28942600 -1.67823900
H 1.51190100 3.70091500 2.17050000
H 2.36036300 5.61863900 0.77690300
C -1.36934500 -0.52464000 -0.69838200
C -2.48263500 -1.24343500 0.10987700
C -3.73352100 -0.42887200 -0.23571800
C -3.56390700 0.78665700 0.44439600
C -2.21422600 0.62691100 1.13518400
C -1.21700900 0.69917200 -0.02363600
H -1.33012600 -0.61486900 -1.79044300
H -1.00820500 1.65025200 -0.52124200
H 0.60825700 -1.55875100 -2.48298500
C 1.33248600 -1.89360700 -1.73010700
C 2.75745400 -1.51780500 -2.00897700
C 0.89214400 -2.77287500 -0.77643800
C 2.08157800 -2.88025200 1.48267200
C 1.78504400 -3.56432800 0.14378300

```
H -0.15940000 -3.07084300 -0.82480500
C 2.21704600 -1.38266700 1.41585700
C 3.61296900 -1.30083800 -0.75891800
C 2.87718500 -0.67084200 0.40514000
H 2.09421100 -0.87639200 2.38088700
H 3.20262300 0.34411300 0.66685800
O 1.26390400 1.00506100 -1.43664800
C 0.86081000 1.23763500 1.29709500
O 0.65652700 1.26668000 2.48863400
H -2.00142200 1.23207200 2.02556900
H 2.73671300 -0.58739500 -2.59549300
H 3.21731500 -2.28721300 -2.65884700
H 4.46011600 -0.64974300 -1.02536200
H 4.06978700 -2.24751900 -0.42267400
H 3.00579600 -3.30047500 1.92459600
H 1.27630300 -3.11109600 2.20004700
H 1.31035500 -4.53850300 0.34330700
H 2.72324800 -3.79982100 -0.38542600
Ir 0.66798000 -0.45625500 0.10528400
H 0.00315300 -1.29308000 1.26437000
C -2.60436100 -2.73620300 0.08260200
H -2.67256000 -3.10505300 -0.95364400
H -1.73400400 -3.20222500 0.57034100
H -3.50826000 -3.05392100 0.62443500
C -4.49973200 1.79584700 0.34921400
C -5.64121600 1.55846600 -0.43622500
C -5.81086100 0.35222200 -1.10735900
C -4.84406000 -0.66459700 -1.01950700
H -4.37080200 2.74844100 0.87162800
H -6.40885500 2.33346200 -0.51832700
H -6.70981000 0.19155400 -1.70966300
H -4.98076500 -1.61212500 -1.55153100
O -2.23653000 -0.76060700 1.43106300
```

1ats2a

Electronic energy at 338.15 K (1,4-dioxane): -1336.591381 au

Electronic energy + thermal free energy correction at 338.15 K (1,4-dioxane): -1336.193023 au

Imaginary frequency (at Def2SVP): -186.12 cm^{-1}

```
C -1.16730800 2.24078600 -0.64279000
C -1.41889600 3.45616000 -1.32338000
C -0.81208600 2.31102700 0.72612400
C -1.28826500 4.66445700 -0.65872300
H -1.69840300 3.41186700 -2.38059500
C -0.69148800 3.54253200 1.38329600
C -0.92052900 4.72483400 0.69707400
H -1.47467600 5.59504200 -1.20579100
H -0.40454400 3.53606000 2.43981300
H -0.82125400 5.69119600 1.19905400
C 1.31175600 -1.23926500 0.43495900
C 2.25464600 -0.39693100 1.30786200
C 3.61787000 -0.65726700 0.67965100
C 3.56339500 0.04640400 -0.53344600
C 2.17481000 0.69875200 -0.54863000
C 1.21995100 -0.49617700 -0.76540700
H 1.37470700 -2.33034100 0.48717800
H 2.14886300 -0.47722300 2.39734300
H 1.17308600 -0.95396400 -1.76096600
H -0.83676700 -1.50630000 -2.56152800
C -1.62247300 -1.78022700 -1.84677600
C -2.95537900 -1.12992500 -2.08533500
C -1.35560000 -2.76960500 -0.95037700
C -2.63817600 -2.82219300 1.28713800
C -2.40458600 -3.43911000 -0.09830300
```

```
H -0.35806400 -3.22706600 -0.97665100
C -2.55361600 -1.32298900 1.36704300
C -3.78528400 -0.82397400 -0.83122700
C -3.01666800 -0.42539100 0.41419400
H -2.35166600 -0.92186600 2.36780400
H -3.15400400 0.60990400 0.75261200
O -1.24108500 1.08669000 -1.24464100
C -0.55025600 1.03457500 1.41865800
O -0.37016900 0.92217000 2.61142400
H -2.76234700 -0.18775400 -2.61774700
H -3.55084100 -1.75878000 -2.77533800
H -4.47661800 -0.00218000 -1.07528600
H -4.43475600 -1.67956000 -0.57640000
H -3.63297300 -3.13114100 1.66364100
H -1.91263100 -3.23915600 2.00538700
H -2.12560300 -4.49608900 0.04019700
H -3.35050900 -3.46377900 -0.66408600
Ir -0.74913200 -0.50780700 0.13122200
H -0.40152300 -1.50752800 1.34246800
O 2.00445700 0.92086100 0.85609900
C 1.98010600 1.95121800 -1.36769200
H 1.10930600 1.86702400 -2.03262800
H 1.80237600 2.80078200 -0.69076000
H 2.86803800 2.18484000 -1.97369800
C 4.72500800 -1.40310900 1.02940900
C 5.81039200 -1.42299100 0.13683700
C 5.75785500 -0.72696900 -1.06606300
C 4.61937900 0.01715900 -1.42112100
H 4.77027000 -1.95426800 1.97371000
H 6.70961000 -1.99078800 0.39321000
H 6.61591500 -0.75593200 -1.74412100
H 4.58517500 0.55971000 -2.37183800
```

1bts2b

Electronic energy at 338.15 K (1,4-dioxane): -1336.594620 au

Electronic energy + thermal free energy correction at 338.15 K (1,4-dioxane): -1336.195914 au

Imaginary frequency (at Def2SVP): -176.30 cm^{-1}

```
C -1.21563400 2.29706700 -0.72608500
C -1.45118600 3.50812900 -1.42014600
C -0.81476100 2.38006900 0.62995000
C -1.28212800 4.72219300 -0.77454200
H -1.76291800 3.45551800 -2.46767300
C -0.65827900 3.61741600 1.26821000
C -0.88403500 4.79411300 0.57188500
H -1.46359000 5.64869300 -1.33018600
H -0.34849000 3.61920600 2.31824600
H -0.75552600 5.76516200 1.05789100
C 1.33679900 -1.16955200 0.17820800
C 2.32575300 -0.42656500 1.09775900
C 3.63916300 -0.54207600 0.31801800
C 3.48251400 0.32490800 -0.77451700
C 2.07681700 0.88731400 -0.58984500
C 1.14526000 -0.29036000 -0.91696600
H 1.41464300 -2.25854700 0.09225700
H 1.05603000 -0.63080900 -1.95493800
H -0.97704000 -1.40091500 -2.65874600
C -1.70119700 -1.72787400 -1.90210900
C -3.07564300 -1.13528400 -2.03144700
C -1.32926500 -2.72119500 -1.05035600
C -2.41963600 -2.86776800 1.27557200
C -2.27893300 -3.45164700 -0.13560100
H -0.31575000 -3.12931300 -1.15568700
C -2.39555400 -1.36611400 1.37512200
```

```
C -3.82558100 -0.89261200 -0.71459200
C -2.98314200 -0.47462900 0.47707200
H -2.15765600 -0.97594300 2.37235200
H -3.17081200 0.53804400 0.85859900
O -1.36183700 1.13385700 -1.29629700
C -0.58476000 1.10629900 1.33764000
O -0.44266200 1.00478300 2.53671500
H -2.96383900 -0.17547600 -2.55608200
H -3.68921900 -1.77836600 -2.69191900
H -4.57344200 -0.10386200 -0.89200000
H -4.41214700 -1.78376800 -0.43037300
H -3.36133300 -3.23127200 1.73165000
H -1.61413400 -3.26007100 1.91924000
H -1.94765800 -4.49875600 -0.04439700
H -3.26602500 -3.50339900 -0.62368600
Ir -0.77164800 -0.43803200 0.05077700
H -0.19032100 -1.40967400 1.21361400
O 2.00132500 0.93643900 0.82620100
C 4.78823400 -1.28581500 0.48987700
C 5.81205600 -1.13749000 -0.46152500
C 5.65822800 -0.27941400 -1.54537200
C 4.47765200 0.46368300 -1.72058500
H 4.91103000 -1.96194200 1.34261600
H 6.74277200 -1.70067100 -0.34643500
H 6.47041700 -0.17773400 -2.27123200
H 4.36479100 1.13533500 -2.57696200
H 1.84163000 1.86075400 -1.04141800
C 2.32744100 -0.74185300 2.56095400
H 1.34656400 -0.49895700 2.99776700
H 2.54408700 -1.81075000 2.71866600
H 3.09912500 -0.15030800 3.07657200
```

2a

Electronic energy at 338.15 K (1,4-dioxane): -1336.607761 au

Electronic energy + thermal free energy correction at 338.15 K (1,4-dioxane): -1336.205945 au

```
C -1.62882900 2.07438600 -0.65580500
C -2.11843400 3.19625600 -1.35852100
C -1.25913600 2.23770800 0.69714700
C -2.20217800 4.42224200 -0.71647600
H -2.40742000 3.07611000 -2.40645900
C -1.35817500 3.48206000 1.33215900
C -1.82480100 4.58103900 0.62808500
H -2.57146100 5.28869700 -1.27526400
H -1.05149500 3.55598600 2.38011600
H -1.90028300 5.56044300 1.10802200
C 1.51286100 -1.10150000 0.64301900
C 2.44014700 -0.11632300 1.40847200
C 3.77013900 -0.24600500 0.69096900
C 3.55410500 0.41775400 -0.52333500
C 2.11707300 0.92565200 -0.44757200
C 1.24007900 -0.33086700 -0.61686400
H 1.90786800 -2.12354200 0.52779800
H 2.42704400 -0.18116400 2.50434300
H 1.39732300 -0.85689900 -1.57211500
H -0.70234600 -1.48654600 -2.58812400
C -1.36227500 -1.93587400 -1.83494100
C -2.82521200 -1.63128300 -1.99477600
C -0.81524400 -2.82712500 -0.95945200
C -1.83462300 -3.11132500 1.36453900
C -1.61126700 -3.70648400 -0.02983200
H 0.25792400 -3.03931600 -1.05470100
C -2.11385900 -1.62756700 1.40446200
C -3.62888400 -1.51219200 -0.69149100
C -2.89306100 -0.90573600 0.48849400
```

```
H -1.99161100 -1.17879900 2.39933900
H -3.29227300 0.05219100 0.84800300
O -1.49337200 0.90577900 -1.23277700
C -0.75866600 1.02635100 1.36837800
O -0.50087900 0.92788400 2.54743000
H -2.89785200 -0.68131600 -2.54438800
H -3.28662500 -2.39762500 -2.64750800
H -4.51769900 -0.89535600 -0.89822700
H -4.03001700 -2.49591100 -0.39113900
H -2.65873600 -3.65022800 1.87199400
H -0.94210800 -3.30265100 1.98747000
H -1.09219000 -4.67270800 0.07875900
H -2.57715000 -3.94731100 -0.50287700
Ir -0.72113200 -0.51260800 0.08045400
H 0.54365800 -1.28819200 1.29151300
C 1.79206400 2.14514700 -1.25643700
H 1.74865700 1.88777000 -2.32681200
H 0.81871200 2.56511300 -0.96553500
H 2.56279600 2.91809900 -1.11245300
C 5.98169500 -0.80292500 -0.00606400
C 5.76869700 -0.14298400 -1.21368700
C 4.53862100 0.47328000 -1.49193900
C 4.97100400 -0.87178100 0.96572600
H 6.95136500 -1.27003400 0.18963900
H 6.57379100 -0.09992200 -1.95322700
H 4.37538800 0.99013200 -2.44359000
H 5.14259500 -1.39481100 1.91163300
O 1.99778100 1.14973700 0.96326000
```

2b

Electronic energy at 338.15 K (1,4-dioxane): -1336.608532 au

Electronic energy + thermal free energy correction at 338.15 K (1,4-dioxane): -1336.206909 au

```
C -1.39126100 2.22153400 -0.74381900
C -1.73390100 3.38512300 -1.46591100
C -1.00931000 2.35848400 0.60767100
C -1.67372900 4.62054800 -0.84003900
H -2.03446700 3.28628200 -2.51287600
C -0.95982200 3.61398600 1.22620400
C -1.28743700 4.75127900 0.50509000
H -1.93397100 5.51724500 -1.41245100
H -0.65375500 3.66390600 2.27584300
H -1.24742700 5.73912200 0.97189600
C 1.47122500 -1.16261400 0.46681500
C 2.48897800 -0.22554800 1.18726000
C 3.73953100 -0.31779500 0.32276100
C 3.42958500 0.41342500 -0.83198800
C 2.00516000 0.88157700 -0.59598600
C 1.12355600 -0.36435600 -0.75556600
H 1.81529600 -2.19715100 0.30682300
H 1.21376700 -0.86593500 -1.73122700
H -0.91341400 -1.42638000 -2.63052600
C -1.58443700 -1.81048500 -1.85178800
C -3.02349100 -1.39726700 -1.98333300
C -1.08379900 -2.73394500 -0.97830600
C -2.08022600 -2.93451500 1.36534100
C -1.92180700 -3.55175800 -0.02803300
H -0.03435400 -3.03187400 -1.10492600
C -2.23514500 -1.43205100 1.39716600
C -3.78708600 -1.21980900 -0.66339500
C -2.97795400 -0.65763900 0.49044500
H -2.05294400 -0.98538600 2.38351900
H -3.29454100 0.33174300 0.84785300
O -1.42236200 1.03262900 -1.29608600
C -0.68708800 1.10125100 1.30258100
```

```
O -0.47809300 0.98835300 2.49048300
H 1.66856100 1.78932100 -1.11673300
H -3.03438200 -0.44326500 -2.53161100
H -3.55420100 -2.12538400 -2.62719200
H -4.63860000 -0.54701200 -0.85188200
H -4.24328700 -2.17402000 -0.34694100
H -2.93826700 -3.40346400 1.88608700
H -1.19734800 -3.19257900 1.97769200
H -1.46778200 -4.55054600 0.07737300
H -2.90962200 -3.72899800 -0.48352600
Ir -0.80072100 -0.45103900 0.02643800
H 0.54473400 -1.30411600 1.18020900
C 2.62792300 -0.36824200 2.67128400
H 2.96722000 -1.38386000 2.93025300
H 3.36358300 0.35457300 3.05536200
H 1.65864300 -0.17701200 3.15604800
C 5.88723400 -0.83562600 -0.57502200
C 5.57968800 -0.11302800 -1.72482100
C 4.33401100 0.51696500 -1.87250900
C 4.95611700 -0.95556400 0.46879300
H 6.86780200 -1.31172400 -0.48259100
H 6.32316300 -0.03035300 -2.52320300
H 4.09974100 1.08096800 -2.78050100
H 5.19872400 -1.52743100 1.37087100
O 2.00590500 1.06273600 0.81501400
```

2ats3a

Electronic energy at 338.15 K (1,4-dioxane): -1336.593325 au

Electronic energy + thermal free energy correction at 338.15 K (1,4-dioxane): -1336.188694 au

Imaginary frequency (at Def2SVP): -199.36 cm $^{-1}$

```
C -0.67522000 2.44021800 -0.92280000
C -0.76625900 3.71308500 -1.52113200
C -0.31896500 2.36798800 0.44280100
C -0.55446400 4.85409900 -0.76007700
H -1.03143700 3.77148200 -2.58082700
C -0.13251000 3.52753800 1.20157000
C -0.24772400 4.77679400 0.60597900
H -0.64180300 5.83565400 -1.23763800
H 0.11523800 3.42014800 2.26240100
H -0.09761300 5.68774300 1.19156100
C 1.29103900 -0.62755900 -1.23096600
C 2.25373100 -1.78434600 -0.88554500
C 3.61877800 -1.16914900 -0.71668700
C 3.52702600 -0.50320200 0.50780100
C 2.12378600 -0.79383400 1.02719200
C 1.20688800 0.12749600 0.14481000
H 1.65009500 0.03738600 -2.03001100
H 2.16840100 -2.67103400 -1.52993500
H 1.70621300 1.09572500 -0.01654900
H -3.11130000 1.33592800 -0.09047800
C -3.01969800 0.26098600 0.12698900
C -3.39097800 -0.11252500 1.53830400
C -3.09060100 -0.61245800 -1.00888900
C -2.42833800 -3.02465600 -0.72265600
C -3.58552900 -2.04536700 -0.92657900
H -3.21617100 -0.11509100 -1.98099600
C -1.30003700 -2.44674200 0.09622100
C -2.76925400 -1.44084100 1.97141700
C -1.44782600 -1.70949300 1.27972500
H -0.31798700 -2.91869000 -0.04643500
H -0.56328900 -1.65664200 1.92147300
O -0.94490800 1.34019600 -1.58646800
C -0.09925500 1.02621100 1.05163200
```

```
O -0.14825900 0.83304100 2.24642500
H -3.02393500 0.69368300 2.19575400
H -4.49171300 -0.12661400 1.65643200
H -2.60029000 -1.42953600 3.05959600
H -3.45907300 -2.28197600 1.78529000
H -2.77635800 -3.97154000 -0.26533400
H -2.01412500 -3.30617400 -1.70659800
H -4.12483100 -2.30161100 -1.85211200
H -4.33174200 -2.14035600 -0.11916000
Ir -1.09529600 -0.34305100 -0.39385100
H 0.36123500 -1.09071900 -1.66721000
C 2.04097600 -0.84703700 2.52633700
H 2.20559700 0.14957600 2.96059300
H 1.06968000 -1.20078900 2.88928500
H 2.83028400 -1.52615700 2.88566700
C 5.74839500 0.31747400 0.22159100
C 5.83814600 -0.34704900 -1.00078500
C 4.76075500 -1.09446100 -1.49473900
C 4.57923600 0.25542600 0.99166400
H 6.60492100 0.89201900 0.58615300
H 6.76392300 -0.28603500 -1.58027600
H 4.83292400 -1.60721100 -2.45876700
H 4.51225800 0.78448300 1.94793600
O 1.88642500 -2.08702300 0.45099300
```

2bts3b

Electronic energy at 338.15 K (1,4-dioxane): -1336.602791 au

Electronic energy + thermal free energy correction at 338.15 K (1,4-dioxane): -1336.197620 au

Imaginary frequency (at Def2SVP): -179.71 cm $^{-1}$

```
C -0.93969200 2.34694300 -1.05524900
C -1.19937000 3.55077900 -1.74125300
C -0.58779900 2.42039500 0.31333100
C -1.15710000 4.76012600 -1.06232000
H -1.45922400 3.49744700 -2.80249300
C -0.57176200 3.64593100 0.98750200
C -0.85643200 4.82166100 0.30609800
H -1.37614000 5.68412800 -1.60764500
H -0.31676500 3.64913500 2.05196900
H -0.84066900 5.78318900 0.82612300
C 1.41345100 -0.51754000 -1.10557800
C 2.45553000 -1.54406900 -0.58041100
C 3.73428700 -0.77400300 -0.31230800
C 3.47315400 -0.04575200 0.85418700
C 2.04566800 -0.40143100 1.19562600
C 1.17571200 0.36001600 0.16947100
H 1.76000800 0.09247100 -1.95315700
H 1.61269300 1.34538200 -0.05978200
H -3.22836500 1.07845600 -0.19750700
C -3.03239300 0.04932300 0.13928900
C -3.37112200 -0.19735100 1.58564100
C -3.00529800 -0.94882400 -0.88966900
C -2.11160600 -3.23549100 -0.34429300
C -3.35872400 -2.40510400 -0.64845300
H -3.16903800 -0.57772100 -1.91148900
C -1.05438700 -2.45946000 0.40052100
C -2.62254900 -1.39992600 2.16214600
C -1.27721200 -1.61354100 1.49664100
H -0.03032600 -2.84262800 0.31013100
H -0.40666700 -1.39769500 2.12554500
O -1.05156100 1.17838800 -1.64173000
C -0.16980700 1.17498000 1.00983900
O -0.13727400 1.06981100 2.21903200
H 1.71645400 -0.29586500 2.23736800
```

```
H -3.09348300 0.71210800 2.14553800
H -4.46565500 -0.30830700 1.71038700
H -2.46150800 -1.24939700 3.24102900
H -3.22508500 -2.32052900 2.07597200
H -2.36602100 -4.15803800 0.21338400
H -1.66201600 -3.57808700 -1.29357200
H -3.86612500 -2.81231300 -1.53719900
H -4.09554200 -2.48335300 0.16936600
Ir -1.05213600 -0.41034600 -0.31361600
H 0.55615800 -1.11277900 -1.52552500
C 2.56330700 -2.82562900 -1.35059400
H 1.58270200 -3.32197300 -1.43232800
H 2.93048200 -2.62772800 -2.37002300
H 3.26581400 -3.51239300 -0.85448400
C 5.64680800 0.92678300 0.72644100
C 5.90773800 0.19977200 -0.43401500
C 4.94148400 -0.65799000 -0.97796300
C 4.41284400 0.82223800 1.38248600
H 6.41889000 1.58699800 1.13232900
H 6.88070300 0.29790000 -0.92419800
H 5.14858900 -1.22070300 -1.89439400
H 4.20949400 1.40291600 2.28717000
O 1.97217600 -1.75207000 0.75060000
```

3a

Electronic energy at 338.15 K (1,4-dioxane): -1336.660158 au

Electronic energy + thermal free energy correction at 338.15 K (1,4-dioxane): -1336.254592 au

```
Ir 1.94172800 -0.09687100 -0.02130000
C -2.40622500 0.46400900 0.00548300
C 3.91270400 -0.16662500 0.73641700
C -2.74976800 -0.99921800 -0.45126000
C -0.95310500 0.86852300 -0.07154600
C -2.83010700 0.40319900 1.50162500
H -3.03119800 1.16570300 -0.56497900
C 1.88897100 -2.06065300 0.72376200
C 2.09696100 -2.10371900 -0.67954900
C 3.93777200 -0.03396500 -0.67559800
O 1.84330300 1.95917600 -0.03480300
C 4.22891900 -1.46051200 1.46851300
H 4.07520800 0.75693000 1.31222500
C -4.26934300 -1.00558800 -0.51938200
C -0.58125300 2.26450900 -0.07146400
O -0.14044900 -0.08322600 -0.05796700
C -3.40504700 -1.02642400 1.59693200
H -3.55405700 1.18483100 1.77976300
H -1.95282700 0.49749600 2.16245400
C 2.96200200 -2.26949800 1.76544200
H 0.86123100 -2.26133400 1.06759500
C 3.43343400 -2.42471600 -1.32769900
H 1.21253900 -2.33355000 -1.29359500
C 4.22538700 -1.15316600 -1.64729300
H 4.13136700 0.97651300 -1.06641300
C 0.79560500 2.70760000 -0.05700800
H 4.75075500 -1.22899200 2.41112200
H 4.94648200 -2.05611400 0.87794300
C -4.69433300 -1.03795600 0.81195800
C -1.60439800 3.25750900 -0.05359900
H -3.42222900 -1.45803400 2.60697500
H 2.54371800 -1.94739700 2.73505000
H 3.19777700 -3.34575300 1.88304200
H 3.26385100 -2.99769100 -2.25361100
H 4.01431000 -3.09833000 -0.67404000
H 5.31243900 -1.36265200 -1.69579900
H 3.94546600 -0.79283000 -2.65263400
C 1.02840300 4.11612400 -0.06026700
C -1.33777500 4.60371300 -0.04345200
H -2.64998700 2.94315400 -0.04022600
C 0.00650900 5.03111000 -0.05341100
H 2.07669800 4.42702600 -0.06024400
H -2.15421400 5.32954600 -0.02971900
H 0.23890300 6.10079300 -0.05229300
C -2.05161900 -1.59064500 -1.64225200
H -2.12389100 -0.90840800 -2.50504900
H -0.99011200 -1.77120700 -1.43163700
H -2.52932700 -2.54395200 -1.91588600
C -6.54068600 -0.94509000 -1.24501200
C -6.96518400 -0.97580800 0.08271300
C -6.04025800 -1.01101500 1.13500600
C -5.17597900 -0.94789100 -1.56342100
H -7.28358700 -0.92366500 -2.04778400
H -8.03633200 -0.97788800 0.30525600
H -6.38070100 -1.02896800 2.17498700
H -4.84634500 -0.91773600 -2.60744600
O -2.52767000 -1.74985400 0.74542000
```

3b

Electronic energy at 338.15 K (1,4-dioxane): -1336.661951 au

Electronic energy + thermal free energy correction at 338.15 K (1,4-dioxane): -1336.257141 au

```
Ir 1.93755100 -0.14064700 -0.10898400
C -2.33450300 0.60824600 -0.39204500
C 3.69430500 -0.44779300 1.01649000
C -2.61015700 -0.88538900 -0.70846600
C -0.86719000 0.96907400 -0.46218600
C -2.83613400 0.67965000 1.07692100
H -2.92650300 1.25827100 -1.05555700
C 1.68709200 -2.19505900 0.24591700
C 2.18325600 -2.00956200 -1.07142500
C 4.02382900 -0.07932100 -0.31432900
O 1.87872400 1.87504200 0.31174000
C 3.82680000 -1.86287400 1.55401100
H 3.75002000 0.35478000 1.76715700
C -4.10882000 -1.01588600 -0.85470400
H -1.97417100 -1.32099200 -1.48915800
C -0.44846700 2.33561800 -0.25297700
O -0.08797400 0.00303600 -0.61785300
C -3.34084500 -0.76682600 1.30950200
H -3.61375400 1.44325700 1.23591200
H -2.00478000 0.88802800 1.77252900
C 2.51232100 -2.64013600 1.42988400
H 0.60633300 -2.38566700 0.34275200
C 3.62122000 -2.29173900 -1.47516000
H 1.44135000 -2.07692000 -1.88183700
C 4.48868100 -1.03140200 -1.39011300
H 4.31570700 0.97045000 -0.47105900
C 0.89644200 2.68993100 0.13515100
H 4.13758900 -1.82589600 2.61063400
H 4.64507000 -2.38571800 1.02888300
C -4.58466200 -0.93249800 0.45873500
C -1.42079000 3.37205600 -0.34602500
H 1.90246000 -2.47089600 2.33445400
H 2.69867000 -3.73179500 1.39395300
H 3.64459900 -2.68744400 -2.50347300
H 4.03599500 -3.09843200 -0.84596100
H 5.55673100 -1.29121900 -1.24923400
H 4.43735800 -0.48875200 -2.35021500
C 1.16194100 4.07184700 0.37240800
C -1.12247400 4.69292600 -0.11906200
H -2.44404500 3.11310900 -0.62865200
```

```
C 0.19476400 5.03774600 0.24847700
H 2.18632000 4.32000100 0.66357500
H -1.89239300 5.46121400 -0.22201500
H 0.45065300 6.08488800 0.43906500
C -3.39741000 -1.22869700 2.73353900
H -4.10817900 -0.61510500 3.30954600
H -3.72175600 -2.27939900 2.78496100
H -2.40546600 -1.14203800 3.20331900
C -6.35332800 -1.17564500 -1.64975500
C -6.82620400 -1.09466600 -0.34097800
C -5.94000200 -0.95966100 0.73646800
C -4.97974300 -1.12469200 -1.92517000
H -7.06499300 -1.28564600 -2.47335800
H -7.90297200 -1.14184500 -0.15332400
H -6.31593900 -0.88962200 1.76279700
H -4.61630200 -1.18264200 -2.95584700
O -2.38182500 -1.51003700 0.54752200
```

Proda

Electronic energy at 338.15 K (1,4-dioxane): -1520.296221 au

Electronic energy + thermal free energy correction at 338.15 K (1,4-dioxane): -1520.066060 au

```
C 0.53356200 0.17222200 -0.21548400
C 1.46765500 -0.86682000 0.49982300
C -0.95659100 -0.14855200 -0.15904700
C 1.05580900 0.06329000 -1.67134900
H 0.73751600 1.16313200 0.21324100
O -3.82489200 -0.44544500 0.39967100
C 2.81211700 -0.16057300 0.57369200
C -1.90997700 0.94791300 -0.06808100
O -1.27780300 -1.34030400 -0.23277400
C 2.23442200 -0.92053000 -1.50524000
H 1.35720500 1.03142400 -2.10147400
H 0.29169900 -0.38504300 -2.32667400
C -3.32109900 0.69576100 0.21038600
C 3.31374400 -0.20221400 -0.73146700
C -1.48099900 2.28471200 -0.28308000
H 2.53878100 -1.45263400 -2.41755300
C -4.16117400 1.86645600 0.27860400
C -2.33287500 3.36554100 -0.23693400
H -0.43190700 2.47429700 -0.53003700
C -3.69419400 3.13762700 0.05976800
H -5.21627800 1.67956600 0.50594200
H -1.96190100 4.37630400 -0.42671400
H -4.38666000 3.98571800 0.11348200
C 4.73467900 1.06830800 1.27284400
C 5.23408700 1.02847000 -0.02810100
C 4.51788300 0.39862600 -1.05544000
C 3.50191400 0.48026300 1.58801900
H 5.31492200 1.56240400 2.05790200
H 6.20049500 1.49177500 -0.24848300
H 4.91222600 0.37770600 -2.07637200
H 3.10906700 0.52332500 2.60958500
O 1.72340000 -1.82258800 -0.53345900
K -3.33172600 -2.80165700 -0.02878700
C 0.99245800 -1.50306500 1.77226700
H 0.74814300 -0.72422000 2.51333400
H 0.09268400 -2.10507300 1.59423500
H 1.78263200 -2.14510200 2.19195400
```

Prodb

Electronic energy at 338.15 K (1,4-dioxane): -1520.300902 au

Electronic energy + thermal free energy correction at 338.15 K (1,4-dioxane): -1520.070629 au

```
C -0.44323600 -0.08507200 0.63606900
C 1.06086600 -0.29109500 0.44392800
C -1.21145100 -1.34203900 1.06967800
H -0.62624200 0.75489700 1.32161900
O 3.95121400 -0.31171400 -0.09732000
C -2.50221800 0.71284500 -0.46124700
C 1.88887000 0.89183500 0.25065100
O 1.48850700 -1.44965900 0.42691700
C -2.19959100 -1.55066200 -0.10444500
H -1.72650000 -1.22691500 2.03601200
H -0.52440700 -2.20045500 1.12408600
C 3.32092800 0.77670700 -0.00680700
C -3.20718400 -0.41818900 -0.03309200
C 1.30575400 2.18747200 0.29801600
C 4.03030000 2.02351100 -0.16833800
C 2.02726800 3.34666600 0.12453600
H 0.23128600 2.28715200 0.48061600
C 3.41686400 3.24803400 -0.10830800
H 5.10676200 1.93516300 -0.35121600
H 1.53454000 4.32148400 0.17195300
H 4.01049500 4.15959500 -0.24302300
C -4.42705100 2.06472100 -0.06281500
C -5.13140900 0.93892900 0.36035500
C -4.52078700 -0.32318000 0.39115800
C -3.08922200 1.96582000 -0.47073700
H -4.92716500 3.03765000 -0.08106300
H -6.17601400 1.04181700 0.66918500
H -5.07691400 -1.20269400 0.73356900
H -2.53655800 2.85319900 -0.79657300
O -1.38900600 -1.12804600 -1.21095300
K 3.60794800 -2.72938600 -0.11124600
C -1.11941800 0.18683100 -0.75242400
H -0.50303600 0.74044000 -1.47532000
C -2.72621800 -2.93927300 -0.30227800
H -3.31197900 -3.25555700 0.57568100
H -3.37571100 -2.98751200 -1.19017900
H -1.89367400 -3.64775500 -0.43423200
```

Carbometalation Path

1cts2c

Electronic energy at 338.15 K (1,4-dioxane): -1336.572494 au

Electronic energy + thermal free energy correction at 338.15 K (1,4-dioxane): -1336.170875 au

Imaginary frequency (at Def2SVP): --198.93 cm^{-1}

```
C -1.31299600 0.50037900 0.26637100
C -2.42002100 -0.09102400 1.20305700
C -3.65908200 -0.05595000 0.30893600
C -3.49462000 -1.11375900 -0.59107300
C -2.13770300 -1.67910900 -0.22658600
C -1.12860800 -0.63854200 -0.72572400
H -1.61438000 1.42816500 -0.24527300
H -1.22108800 -0.36674200 -1.78748800
H 0.49890100 -3.18111000 -0.72807900
C 1.39977200 -2.61336500 -0.45974200
C 2.21939000 -3.24079300 0.63656700
C 1.91584200 -1.73646100 -1.44807700
C 3.76045200 -0.08509500 -0.79317200
C 3.37919700 -1.35583300 -1.56383300
H 1.33574200 -1.65480100 -2.37770300
```

```
C 2.98026500 0.12541800 0.47404500
C 3.17115700 -2.26507300 1.33868100
C 2.67078500 -0.84108700 1.40361100
H 2.82560900 1.17129200 0.76935300
H 2.25717400 -0.49946100 2.35988100
H 1.51735400 -3.66665800 1.37277200
H 2.78559100 -4.09982900 0.22760700
H 3.35468900 -2.62056500 2.36483700
H 4.15803800 -2.27158600 0.84672300
H 4.84472400 -0.08948000 -0.57010400
H 3.58739500 0.79387800 -1.43471200
H 3.61943400 -1.20908300 -2.62821800
H 3.99824100 -2.21018300 -1.23944500
O -2.11644700 -1.48602000 1.18252800
C -4.76379800 0.77281000 0.25283600
C -5.73061900 0.50785800 -0.72898600
C -5.56772700 -0.54568700 -1.62530200
C -4.43316800 -1.36949800 -1.57350200
H -4.89278600 1.60430300 0.95372900
H -6.62542100 1.13423500 -0.78929500
H -6.33743300 -0.73478800 -2.37941300
H -4.30853700 -2.19065700 -2.28613700
H -1.93623100 -2.72817900 -0.48795400
Ir 0.78786600 -0.59047000 -0.04674600
C -0.00522700 1.30570500 1.14221500
H 0.33774500 -1.55585900 1.11896000
C 0.55663900 2.43253800 0.38000800
O 0.00243900 1.22530000 2.35120500
C 0.69731000 3.68550900 0.99708000
C 0.96442600 2.22854700 -0.96904900
C 1.16919100 4.77167500 0.28052400
H 0.40643500 3.77836300 2.04859600
C 1.42309300 3.36119600 -1.68792000
O 0.96519800 1.04218800 -1.49482000
C 1.51960300 4.59534400 -1.07017500
H 1.26344900 5.75307900 0.75270000
H 1.72217100 3.21713000 -2.73071500
H 1.89082200 5.45080800 -1.64452500
C -2.64295600 0.40455100 2.60203700
H -2.73550300 1.50197400 2.61654600
H -3.57836600 -0.03083600 2.98700000
H -1.81255000 0.12949300 3.26151900
```

1dts2d

Electronic energy at 338.15 K (1,4-dioxane): -1336.578080 au

Electronic energy + thermal free energy correction at 338.15 K (1,4-dioxane): -1336.176122 au

Imaginary frequency (at Def2SVP): -177.57 cm $^{-1}$

```
C -1.24416700 0.71359400 0.29400900
C -3.63307100 0.46393800 0.55850000
C -3.64505100 -0.64373800 -0.29744400
C -2.34799800 -1.38271000 0.00707300
C -1.24465200 -0.49893500 -0.62186000
H -1.43883400 1.63900700 -0.26868500
H -1.37629700 -0.28592400 -1.69390000
H 0.28590800 -3.12721600 -0.81184300
C 1.21340000 -2.62661500 -0.51062700
C 1.97608000 -3.33701600 0.57397600
C 1.78222800 -1.74499400 -1.45782700
C 3.72433600 -0.23563500 -0.74282900
C 3.26477500 -1.44834100 -1.56070900
H 1.20620100 -1.58528200 -2.37962700
C 2.94914300 -0.02010500 0.52511500
C 2.97908600 -2.44536400 1.31423400
C 2.56869500 -0.99512200 1.41918000
H 2.86523400 1.02201500 0.85779200
H 2.17396000 -0.65776500 2.38495000
H 1.23682900 -3.73672700 1.28942400
H 2.49002100 -4.22006300 0.14751300
H 3.12986500 -2.84231000 2.33043100
H 3.96839300 -2.49900400 0.82990300
H 4.80355400 -0.32290900 -0.51233300
H 3.62018200 0.67571000 -1.35316600
H 3.51377300 -1.27716700 -2.61937500
H 3.82836300 -2.35160700 -1.26923600
O -2.19744300 -1.08569900 1.40218000
C -4.64663800 1.40336400 0.53226700
C -5.70502800 1.19793500 -0.36595300
C -5.71869900 0.09490200 -1.21633300
C -4.67405700 -0.84225400 -1.19867000
H -4.63767300 2.27480600 1.19402000
H -6.53294000 1.91231100 -0.39679300
H -6.55714400 -0.04377500 -1.90530900
H -4.68592100 -1.70456900 -1.87394300
Ir 0.70621800 -0.58036800 -0.01401600
C 0.10938400 1.39124900 1.19383100
H 0.20474800 -1.53244800 1.13958800
C 0.79879800 2.44910600 0.44395500
O 0.04212200 1.32152100 2.40161600
C 1.09165800 3.66983700 1.07265400
C 1.15433000 2.21859000 -0.91653200
C 1.67736700 4.70213900 0.36096500
H 0.82710200 3.78249300 2.12905000
C 1.72870800 3.30085600 -1.63011400
O 1.00314700 1.04943500 -1.45657200
C 1.98140900 4.50536200 -0.99833300
H 1.89424900 5.65899000 0.84299900
H 1.98840000 3.13897000 -2.68081100
H 2.44070200 5.31970100 -1.56875400
C -2.33093100 0.32155700 1.31853800
H -2.24121600 0.79082200 2.30566900
C -2.33657200 -2.86096000 -0.24580700
H -2.27342100 -3.07071300 -1.32601000
H -1.47619900 -3.32890400 0.25631200
H -3.25445700 -3.32598200 0.14599100
```

2c

Electronic energy at 338.15 K (1,4-dioxane): -1336.589749 au

Electronic energy + thermal free energy correction at 338.15 K (1,4-dioxane): -1336.187326 au

```
C -1.72195200 0.69310700 0.29092700
C -3.78077000 -0.55071800 0.25653700
C -3.19842100 -1.51486600 -0.57522000
C -0.96845000 -0.41851000 -0.52351600
H -2.30551700 1.37449500 -0.34769100
H -1.01924500 -0.23911200 -1.61152100
H 0.53872300 -2.68817900 -1.28298100
C 1.47440700 -2.27440500 -0.88341500
C 2.27756800 -3.25189700 -0.06068700
C 2.02418700 -1.17372600 -1.58469500
C 4.02355600 0.06150100 -0.54219400
C 3.50905800 -0.86474000 -1.65450900
H 1.42550400 -0.77822700 -2.41682300
C 3.25815600 -0.04039500 0.74869600
C 3.30790000 -2.58808700 0.86191100
C 2.89851900 -1.22124000 1.36796200
H 3.16387400 0.88705600 1.32837700
H 2.54305200 -1.15795000 2.40368900
H 1.56416200 -3.83679100 0.54545200
```

```
H 2.77318800 -3.98221300 -0.72889300
H 3.49333700 -3.24592900 1.72571700
H 4.28049700 -2.49975700 0.34925700
H 5.09731500 -0.13278200 -0.35617100
H 3.94608400 1.10381500 -0.88197900
H 3.71949900 -0.39546800 -2.62796200
H 4.06803500 -1.81710700 -1.66198600
O -1.96317600 -1.38737100 1.33051100
C -5.07867700 -0.11517200 0.05771500
C -5.80845500 -0.69224900 -0.99096100
C -5.22979900 -1.65077000 -1.82179100
C -3.90551900 -2.06703200 -1.62994000
H -5.53226800 0.64680600 0.70058000
H -6.84604800 -0.38895300 -1.15902900
H -5.82108200 -2.08744100 -2.63216200
H -3.45645700 -2.81563700 -2.29029800
Ir 1.06555800 -0.46908800 0.16479500
C -0.57649600 1.44142600 0.89023800
H 0.56553700 -1.67869400 1.02328000
C -0.20622100 2.71251000 0.28916000
O 0.23124500 0.86622000 1.66463600
C -0.96742900 3.88447300 0.37779000
C 0.95461400 2.62742200 -0.54825800
C -0.52977500 5.05104700 -0.22885200
H -1.88811800 3.87701700 0.97397900
C 1.37904500 3.84674400 -1.14336800
O 1.51812000 1.50435400 -0.83712900
C 0.65887300 5.01549200 -0.97840100
H -1.09362400 5.98109800 -0.12210700
H 2.28072500 3.81776800 -1.76314700
H 1.01578300 5.93163400 -1.46103600
C -2.66569200 -0.15246300 1.20430200
C -1.78678600 -1.65312000 -0.05618600
H -1.30809500 -2.63136000 -0.20823800
C -3.03339200 0.43622400 2.53236500
H -2.13464700 0.60681700 3.14412200
H -3.54871100 1.40062300 2.39085800
H -3.70725000 -0.24067000 3.07963600
```

2d

Electronic energy at 338.15 K (1,4-dioxane): -1336.591512 au

Electronic energy + thermal free energy correction at 338.15 K (1,4-dioxane): -1336.188087 au

```
C 1.56176500 1.46288700 0.15345100
C 3.94420100 0.80828400 0.08658700
C 3.61469800 -0.53884000 -0.10546300
C 2.34138900 -0.51382200 -0.93858500
C 1.24296700 -0.05272600 0.09763600
H 1.64536100 1.83762400 1.18904400
H 1.46264500 -0.52162800 1.07442600
H 0.54677700 -2.67492700 -0.29586900
C -0.52112100 -2.48963500 -0.15335300
C -1.41361500 -3.17265400 -1.15425500
C -0.91331900 -2.10499400 1.14326800
C -3.28152200 -1.26156200 1.57179800
C -2.27213200 -2.39595700 1.75023100
H -0.10303700 -1.94157700 1.86687000
C -3.15127600 -0.52758900 0.26578600
C -2.82111000 -2.57401400 -1.24328100
C -2.89159800 -1.08702000 -0.97061700
H -3.54297900 0.49510300 0.26568600
H -3.05213100 -0.43813500 -1.84050400
H -0.92088700 -3.10410500 -2.13947700
H -1.47057600 -4.25488000 -0.92693600
H -3.22953600 -2.76539400 -2.24817000
H -3.50402000 -3.09227200 -0.54934900
H -4.31460500 -1.64056400 1.69616600
H -3.13795700 -0.52262300 2.37865800
H -2.13914200 -2.59179100 2.82570500
H -2.65969900 -3.33788800 1.32620500
O 2.55967300 0.66543700 -1.72025400
C 5.02697000 1.17604000 0.86595100
C 5.80133400 0.15764300 1.43979000
C 5.47411800 -1.18334700 1.24737600
C 4.36122900 -1.54862500 0.47665500
H 5.28416800 2.22786100 1.02597100
H 6.67661100 0.41809100 2.04245700
H 6.09675200 -1.96060400 1.70087500
H 4.10452900 -2.60480400 0.33850200
Ir -0.84707700 -0.34158700 -0.10190900
C 0.49081600 2.24264900 -0.58069400
H -0.61336800 -0.65846700 -1.62950800
C -0.94778700 1.94986500 -0.25050900
O 0.75283500 3.06143500 -1.43506300
C -1.88732900 2.61515400 -1.12264700
C -1.36882800 1.75176700 1.14625500
C -3.06551000 3.12998900 -0.67058400
H -1.57906900 2.75137800 -2.16397300
C -2.54160600 2.44058600 1.60963000
O -0.77324300 0.88592200 1.86246600
C -3.35376900 3.09546500 0.72747000
H -3.75757700 3.62780700 -1.35429900
H -2.77532500 2.36479600 2.67572900
H -4.25941200 3.58904700 1.09535900
C 2.86647200 1.55826900 -0.65382900
H 3.09587300 2.56066200 -1.03674100
C 2.15296800 -1.67814000 -1.86745600
H 2.18286800 -2.64161500 -1.33553100
H 1.20420500 -1.60139500 -2.42058300
H 2.97994200 -1.67788000 -2.59456000
```

2cts3c

Electronic energy at 338.15 K (1,4-dioxane): -1336.580487 au

Electronic energy + thermal free energy correction at 338.15 K (1,4-dioxane): -1336.179770 au

Imaginary frequency (at Def2SVP): -681.13 cm^{-1}

```
C -1.40305200 0.75679000 0.29987000
C -2.49410400 0.15424100 1.25107900
C -3.74004800 0.03367300 0.38835900
C -3.51394400 -1.07092600 -0.43605800
C -2.12402600 -1.51881700 -0.05216200
C -1.16490900 -0.47256500 -0.66437900
H -1.83427700 1.57859500 -0.29993500
H -1.48826600 -0.18415000 -1.67787700
H 0.17352200 -1.62463100 1.88088300
C 1.12317900 -1.77285500 1.35079100
C 2.37736900 -1.44484700 2.14066400
C 1.08470800 -2.63663800 0.24256500
C 3.50039400 -2.46699300 -0.52527200
C 2.25661800 -3.34468400 -0.38663200
H 0.12325500 -3.13213200 0.05095700
C 3.16609100 -1.02763400 -0.83547400
C 3.13373500 -0.21560600 1.62059000
C 3.05390500 -0.02639900 0.12618100
H 3.30054200 -0.69919800 -1.87459300
H 3.17035800 1.00837100 -0.22469800
H 2.08837800 -1.26086900 3.18720900
```

```
H 3.03508200 -2.33107800 2.16425200
H 2.70209200 0.68806000 2.08295200
H 4.19170200 -0.24870500 1.94606400
H 4.11198200 -2.50690200 0.39110500
H 4.14123200 -2.86593400 -1.32760700
H 1.93764800 -3.68074800 -1.38871300
H 2.49222900 -4.26797800 0.17752100
O -2.08570300 -1.21835100 1.33512500
C -4.90230100 0.77817000 0.28982000
C -5.85770900 0.37725600 -0.65382200
C -5.63084400 -0.72418900 -1.47807100
C -4.44165400 -1.46008200 -1.38697000
H -5.08217900 1.64744400 0.93117600
H -6.79605200 0.93252700 -0.74286400
H -6.39431600 -1.01970900 -2.20377800
H -4.26634800 -2.31893300 -2.04226500
H -1.87440200 -2.57407200 -0.23485700
Ir 0.97439600 -0.59053100 -0.45473000
C -0.17545200 1.36636000 1.00773600
H -0.05723000 -1.30433600 -1.45179200
C 0.58029700 2.40692200 0.25061500
O 0.06942400 1.15244500 2.18453100
C 0.87026500 3.60650400 0.91776200
C 0.97757600 2.23077700 -1.10376700
C 1.45499000 4.67375000 0.25212700
H 0.59327200 3.68629000 1.97410800
C 1.54730300 3.33741400 -1.77143000
O 0.89318800 1.06897300 -1.70939400
C 1.77369900 4.53137600 -1.10538200
H 1.65472000 5.61284500 0.77485000
H 1.83773700 3.20192200 -2.81766700
H 2.22899000 5.36751800 -1.64630500
C -2.72679600 0.75962200 2.60636500
H -1.85907500 0.61768400 3.25941500
H -2.90937200 1.84261500 2.51180600
H -3.61671600 0.29811600 3.06214800
```

2dts3d

Electronic energy at 338.15 K (1,4-dioxane): -1336.579845 au

Electronic energy + thermal free energy correction at 338.15 K (1,4-dioxane): -1336.179027 au

Imaginary frequency (at Def2SVP): -653.88 cm^{-1}

```
C -1.36838600 0.90369000 0.40284400
C -3.74418200 0.43539100 0.67772900
C -3.65509000 -0.65059700 -0.19632700
C -2.31311700 -1.29987800 0.10893600
C -1.25581500 -0.35473300 -0.53943100
H -1.76462600 1.73972500 -0.20163400
H -1.57710600 -0.06014700 -1.55212700
H 0.10486000 -1.82739400 1.79784600
C 1.07354200 -1.85952000 1.27888200
C 2.28169800 -1.50189200 2.12884200
C 1.14495200 -2.65588000 0.11802100
C 3.58060900 -2.33409200 -0.54366800
C 2.37367900 -3.26530100 -0.50441000
H 0.24044800 -3.21141800 -0.14730400
C 3.19440900 -0.90275100 -0.80196900
C 3.01483000 -0.22888200 1.68313700
C 2.99566400 0.02825300 0.19808700
H 3.30840000 -0.52066200 -1.82475100
H 3.03966200 1.08515600 -0.09558200
H 1.94930700 -1.36219900 3.16905500
H 2.97254800 -2.36319400 2.15177900
H 2.54130000 0.64171400 2.16701100
H 4.06105700 -0.24265700 2.04574400
H 4.14811300 -2.38858900 0.40000300
H 4.27581800 -2.66509800 -1.33162900
H 2.10574200 -3.55088700 -1.53680700
H 2.62802500 -4.21245300 0.01039700
O -2.16641800 -0.99207600 1.49734300
C -4.83841600 1.28295100 0.65603300
C -5.86512100 1.00212900 -0.25606500
C -5.77435900 -0.08134100 -1.12896300
C -4.65339400 -0.92195800 -1.11537600
H -4.91212400 2.14105200 1.33106100
H -6.75284900 1.64098600 -0.28223600
H -6.59102900 -0.27904600 -1.82952400
H -4.58213100 -1.76972300 -1.80545300
Ir 0.89019000 -0.59009300 -0.44837900
C -0.13040400 1.46904100 1.11853700
H -0.21218600 -1.29286900 -1.36516900
C 0.71152800 2.43036000 0.35871300
O 0.02348600 1.29729300 2.31756400
C 1.17904500 3.56697000 1.03651900
C 1.00802000 2.25351900 -1.02242300
C 1.85031200 4.57645900 0.36410000
H 0.97100700 3.64343400 2.10868400
C 1.65476500 3.31171500 -1.69918400
O 0.76920100 1.12248200 -1.63994500
C 2.06429100 4.44532900 -1.01601100
H 2.19339000 5.46727800 0.89679200
H 1.86243000 3.17943800 -2.76526700
H 2.58251100 5.24073000 -1.56181200
C -2.42885700 0.40305100 1.41590900
H -2.36353300 0.87193400 2.40533700
C -2.26613400 -2.78262000 -0.13629200
H -2.05188600 -3.00912800 -1.19353600
H -1.50292600 -3.25769400 0.49484100
H -3.23343800 -3.23920300 0.12276500
```

Endo Path

1e

Electronic energy at 338.15 K (1,4-dioxane): -1336.597344 au

Electronic energy + thermal free energy correction at 338.15 K (1,4-dioxane): -1336.195904 au

```
C -2.90774200 -0.33422700 -0.47475900
C -4.25704000 -0.49537700 -0.88382200
C -2.49517600 -1.02318100 0.70041200
C -5.12260900 -1.28383500 -0.14834700
H -4.58524700 0.02388700 -1.78927900
C -3.39245900 -1.82059200 1.43068100
C -4.70507100 -1.95526100 1.01703100
H -6.16049400 -1.38775200 -0.48299800
H -3.01763600 -2.32566000 2.32701900
H -5.40949900 -2.57319900 1.58030100
C 0.08592400 -1.55999000 -1.00082600
C 1.22974300 -2.55161100 -0.63958300
C 2.29526700 -1.84802800 0.18062600
C 2.90178000 -0.95689400 -0.71508900
C 2.13636200 -1.16634500 -2.00105900
C 0.67590100 -0.63552100 -1.87204400
```

```
H -0.08215100 1.97173700 -2.50314400
C 0.02955500 2.42245700 -1.50976800
C -1.11846300 3.28633000 -1.07685500
C 1.26390200 2.33579800 -0.91234800
C 1.43155900 2.53291300 1.63778300
C 1.71050400 3.16252400 0.26768100
H 2.04770900 1.80256900 -1.46254100
C 0.18413100 1.69413700 1.70824700
C -1.33450500 3.34178500 0.43608000
C -1.05620600 2.03698500 1.15337100
H 0.18242500 0.95785000 2.52174900
H -1.93528600 1.53561600 1.57772800
O -2.07059600 0.40112700 -1.13906000
C -1.09389500 -0.88124600 1.11767300
O -0.59859300 -1.41672400 2.08363900
H 2.64084000 -0.89606200 -2.93861900
H -2.02305500 2.87913800 -1.55348800
H -0.98510600 4.30703300 -1.48454400
H -2.37897600 3.63080900 0.63088300
H -0.72256500 4.13833300 0.89339300
H 1.37395300 3.32201400 2.41218800
H 2.27991300 1.88907500 1.92518700
H 2.79443300 3.34056500 0.17772200
H 1.24196200 4.15858500 0.19741700
Ir -0.10980600 0.47226900 -0.13559900
H 1.28372300 0.23404300 0.53895700
C 0.77663700 -3.90655200 -0.19308600
H 0.23418200 -3.81609200 0.76190300
H 0.09770500 -4.34899500 -0.93817100
H 1.63661300 -4.57869300 -0.05290200
C 3.91663200 -0.11077300 -0.30971100
C 4.32103500 -0.17421300 1.03284000
C 3.70973400 -1.05375100 1.92515900
C 2.67722500 -1.90681600 1.50675700
H 4.39541700 0.58765400 -1.00444200
H 5.13401500 0.46917800 1.38301000
H 4.04641100 -1.08462700 2.96555100
H 2.18346400 -2.58029200 2.21321200
O 1.88991500 -2.57502200 -1.92578300
H -0.92489500 -1.96381900 -1.11856600
H 0.16510400 -0.22497600 -2.74905300
```

1f

Electronic energy at 338.15 K (1,4-dioxane): -1336.596739 au

Electronic energy + thermal free energy correction at 338.15 K (1,4-dioxane): -1336.195032 au

```
C 2.93409000 -0.45264300 0.59740000
C 4.24576300 -0.63200800 1.10825900
C 2.63307500 -1.05769400 -0.65521400
C 5.18209800 -1.35781300 0.39520200
H 4.48842600 -0.17710200 2.07331500
C 3.60058800 -1.79235000 -1.36111900
C 4.87514500 -1.94576600 -0.84705400
H 6.18910300 -1.47721800 0.80955700
H 3.31061600 -2.23380200 -2.32034200
H 5.63392100 -2.51502400 -1.39071500
C -0.09569700 -1.72874700 0.78128300
C -2.21925200 -1.91519000 -0.57310800
C -2.87934700 -1.07083300 0.33052100
C -2.22422600 -1.35880700 1.66791200
C -0.74391600 -0.85060600 1.65934700
H -0.08415600 1.70023800 2.52393200
C -0.10791400 2.21810500 1.55749100
C 1.06991800 3.10798600 1.28933400
C -1.28200700 2.17271600 0.84598600
C -1.22735700 2.53541600 -1.68896100
C -1.62665700 3.07634200 -0.31116300
H -2.10804000 1.59983800 1.28203500
C 0.02976100 1.70874400 -1.70429600
C 1.41964100 3.26681800 -0.19101300
C 1.21343100 2.01501600 -1.01938800
H 0.11041900 1.02969900 -2.56232800
H 2.13016300 1.54612200 -1.39885400
O 2.03296300 0.22666500 1.23757500
C 1.27046800 -0.89656100 -1.18106400
O 0.86412300 -1.36347600 -2.22024600
H 1.93015400 2.66749400 1.81589400
H 0.89637300 4.09866700 1.75222700
H 2.47596400 3.56708000 -0.27130000
H 0.84721800 4.09380600 -0.64535600
H -1.11291800 3.37155100 -2.40545500
H -2.04189200 1.90585600 -2.08576100
H -2.71493800 3.25100000 -0.30451800
H -1.16950300 4.06469200 -0.13631500
Ir 0.16973800 0.36258000 0.07079100
H -1.15608600 0.16834000 -0.74093600
C -3.85942900 -0.19251000 -0.09127100
C -4.16833800 -0.16838700 -1.46033500
C -3.49809700 -0.99576500 -2.36006300
C -2.50398800 -1.88517400 -1.92424500
H -4.38289400 0.46635900 0.61094700
H -4.95086900 0.50328300 -1.82635700
H -3.75886700 -0.95793200 -3.42180100
H -1.96625300 -2.51919300 -2.63421300
O -1.97484400 -2.77204600 1.50946100
H 0.90270200 -2.14308200 0.94521100
H -0.29922200 -0.50221100 2.59915600
C -1.23022900 -2.66473200 0.28992800
H -0.89227200 -3.64564800 -0.06755500
C -2.99772000 -1.06204700 2.91603100
H -3.98172300 -1.55410200 2.88595800
H -2.44995400 -1.42143300 3.80063200
H -3.15204700 0.02384000 3.02535600
```

1ets2e

Electronic energy at 338.15 K (1,4-dioxane): -1336.578695 au

Electronic energy + thermal free energy correction at 338.15 K (1,4-dioxane): -1336.179967 au

Imaginary frequency (at Def2SVP): -172.02 cm^{-1}

```
C 2.77825300 -0.48484400 0.94938100
C 3.82975700 -0.79825300 1.84758300
C 3.02687100 -0.64389900 -0.43812600
C 5.04639100 -1.24160400 1.36179900
H 3.64881800 -0.68100300 2.92013900
C 4.27166900 -1.09280500 -0.91023200
C 5.28435500 -1.39488400 -0.01801400
H 5.84594500 -1.48006100 2.07154000
H 4.40786700 -1.19453900 -1.99167700
H 6.25647800 -1.74702700 -0.37333600
C -0.60684600 -1.49626200 -1.38793200
C -3.02603500 -1.07497700 -0.72558800
C -2.72255600 -1.40132400 0.60291100
C -1.60264100 -2.42352400 0.50510300
C -0.29483000 -1.78985200 -0.04599600
H -1.02727400 0.58297100 2.24178000
C -0.83061400 1.49727700 1.66498800
C 0.27930300 2.37385300 2.16521600
C -1.68890400 1.80267100 0.66102400
C -0.88070200 3.05709400 -1.40161300
```

```
C -1.70224100 3.09048700 -0.10876700
H -2.50414900 1.11003400 0.46533100
C 0.43359500 2.33089700 -1.32041300
C 1.09800900 3.07647700 1.07273300
C 1.29829100 2.31262800 -0.22292500
H 0.87522300 2.08823400 -2.29522300
H 2.34402700 2.06737400 -0.44983800
O 1.62113700 -0.06378600 1.35753100
C 1.92405900 -0.31247800 -1.34905300
O 1.98980800 -0.36057700 -2.55894600
H 0.95204900 1.74477100 2.76457500
H -0.13721700 3.13159900 2.85652300
H 2.09475300 3.29839700 1.48563400
H 0.66184500 4.06174400 0.83265300
H -0.68191000 4.09230500 -1.74127100
H -1.47814500 2.59321100 -2.20562000
H -2.74742600 3.32922300 -0.36832400
H -1.36198900 3.91372400 0.54006600
Ir 0.29137900 0.29466600 -0.30890000
C -3.47532900 -0.90407200 1.65018900
C -4.55518200 -0.06168900 1.33914100
C -4.84512200 0.27461700 0.01851700
C -4.07131400 -0.22768400 -1.04070300
H -3.25632800 -1.16976800 2.68988600
H -5.18201600 0.33100500 2.14485500
H -5.69749500 0.92549200 -0.19692900
H -4.31498700 0.02142400 -2.07807100
O -2.03615400 -3.10420600 -0.70238600
H 0.05928000 -1.62319300 -2.24530200
H 0.64679300 -2.26599600 0.25552200
H -0.71903800 0.37333400 -1.58718200
C -2.08236400 -1.93727900 -1.52949900
H -2.38526900 -2.18142300 -2.55595300
C -1.42273000 -3.36200100 1.65646000
H -0.67456200 -4.13105400 1.41147400
H -1.06611100 -2.80534600 2.53827500
H -2.37254900 -3.85756900 1.90906000
```

1fts2f

Electronic energy at 338.15 K (1,4-dioxane): -1336.578513 au

Electronic energy + thermal free energy correction at 338.15 K (1,4-dioxane): -1336.179952 au

Imaginary frequency (at Def2SVP): -184.03 cm^{-1}

```
C 2.87154200 -0.24026400 1.02798700
C 3.98067800 -0.27726300 1.91068400
C 3.01980400 -0.85196100 -0.24331800
C 5.15538400 -0.89433900 1.52086700
H 3.87739800 0.18907600 2.89492600
C 4.22385700 -1.46998400 -0.62068300
C 5.29394600 -1.49675600 0.25509400
H 6.00063400 -0.91414200 2.21731200
H 4.28348100 -1.92285600 -1.61553900
H 6.23521300 -1.97647900 -0.02624500
C -0.68115500 -1.81671300 -0.64911900
C -2.17945600 -2.21893200 -0.58829300
C -3.03507800 -1.10670200 -0.01583300
C -2.65810300 -1.02954200 1.33158700
C -1.57716800 -2.08047900 1.46700500
C -0.28499100 -1.70272900 0.70032200
H -0.80667500 1.34964200 2.13881800
C -0.63726500 2.01636100 1.28263400
C 0.52885400 2.95397500 1.38878100
C -1.56541800 2.01602100 0.29583800
C -0.89358400 2.47844700 -2.11587000
C -1.60878900 2.97629900 -0.85582700
H -2.40844700 1.33638600 0.39626300
C 0.41170100 1.76715000 -1.89036900
C 1.27654200 3.22079700 0.07446000
C 1.35804400 2.07011900 -0.91072800
H 0.76939100 1.19568300 -2.75616100
H 2.37691600 1.71527800 -1.11309500
O 1.75101700 0.33012900 1.34653200
C 1.85987800 -0.79774800 -1.14102500
O 1.83964500 -1.23214800 -2.27260800
H -1.41391300 -2.49446800 2.46997500
H 1.23143800 2.52593100 2.11711500
H 0.18513500 3.91575200 1.81589700
H 2.30768500 3.51566100 0.32564400
H 0.84849700 4.09495600 -0.44639200
H -0.70570900 3.33184700 -2.79633500
H -1.56097700 1.79903900 -2.67413000
H -2.66501300 3.16683000 -1.11061500
H -1.19838400 3.94989800 -0.54232800
Ir 0.31080900 0.16663700 -0.25506200
C -2.69362200 -2.93665500 -1.79750200
H -2.70514300 -2.25600000 -2.66395400
H -2.04692200 -3.79334700 -2.04044200
H -3.71656900 -3.30272400 -1.62262100
C -3.32922100 -0.20188700 2.21248700
C -4.40483000 0.55143600 1.71427200
C -4.77137600 0.48078600 0.37188500
C -4.07754000 -0.35279100 -0.52065300
H -3.05398900 -0.14788200 3.27034900
H -4.96911800 1.19769100 2.39270200
H -5.61950300 1.07094700 0.01273500
H -4.37876500 -0.42087600 -1.57142900
O -2.10172900 -3.07615900 0.57001700
H -0.08418900 -2.24930600 -1.45874500
H 0.66331700 -2.10491600 1.07443000
H -0.78533800 -0.11711100 -1.42795400
```

2e

Electronic energy at 338.15 K (1,4-dioxane): -1336.604435 au

Electronic energy + thermal free energy correction at 338.15 K (1,4-dioxane): -1336.201054 au

```
C 1.71405600 2.11264400 -0.35419800
C 2.51925900 3.18835700 -0.78695400
C 0.92260500 2.28497300 0.79882500
C 2.50597100 4.37784100 -0.07492100
H 3.13555500 3.06188700 -1.68141600
C 0.92607500 3.49144900 1.51015800
C 1.71514700 4.54478500 1.07507000
H 3.13010600 5.20854100 -0.42094500
H 0.29439300 3.57337200 2.40025300
H 1.72632700 5.49465800 1.61631700
C -1.71786200 1.59589100 -1.39442500
C -3.10345000 1.00222700 -1.06126000
C -2.93634900 0.17315600 0.19625800
C -2.18887500 -0.95716100 -0.21523300
C -2.00704000 -0.72865900 -1.70450100
C -0.89057500 0.32566900 -1.65249900
H 1.22803400 -1.31268300 -2.65948700
C 1.58555300 -1.79869600 -1.74312400
C 3.02846000 -1.55887400 -1.40072500
C 0.76132000 -2.70457400 -1.14379100
C 1.09858700 -3.16389000 1.38465400
C 1.20037300 -3.66685600 -0.06585800
H -0.20811300 -2.89280000 -1.62000600
C 1.28607000 -1.68289800 1.58815100
```

C 3.36378200 -1.53052300 0.09830800
C 2.30634100 -0.94406100 1.00943100
H 0.80064600 -1.26273400 2.47910200
H 2.55630700 0.01653600 1.47775900
O 1.68682700 0.96652000 -0.98456500
C 0.10894100 1.11284500 1.17678900
O -0.56070400 1.05560300 2.18557000
H -1.85269300 -1.60607800 -2.34753600
H 3.31701800 -0.59860600 -1.85129900
H 3.64670800 -2.33025400 -1.89998800
H 4.29172400 -0.95125200 0.22724200
H 3.60685800 -2.54661900 0.45608900
H 1.84533300 -3.69986200 2.00311000
H 0.12582900 -3.45339400 1.81271800
H 0.60584200 -4.59101500 -0.15810700
H 2.23783400 -3.97308900 -0.28301200
Ir 0.37471400 -0.39300900 -0.11477600
C -4.27561200 1.93196100 -1.12609400
H -4.16866400 2.73331300 -0.37788200
H -4.33930300 2.39697000 -2.12195400
H -5.21444100 1.39125000 -0.92836100
C -1.83126900 -1.94681900 0.69792500
C -2.26319400 -1.79781100 2.03514900
C -2.99299700 -0.69176600 2.42288200
C -3.33950500 0.31778200 1.49825800
H -1.37713700 -2.88373500 0.37129100
H -2.02929300 -2.57873300 2.76523300
H -3.31573800 -0.59724900 3.46328500
H -3.91700200 1.18893100 1.82345900
O -3.19095700 -0.03479000 -2.05467400
H -1.81947200 2.19616400 -2.31593000
H -0.28225500 0.39733500 -2.56794800
H -1.33359000 2.26020000 -0.60454600
H -1.72443900 1.67102500 1.53248900
O -1.73607200 0.63973200 -1.05513500
C -1.05218400 -0.80705100 1.10109300
O -0.67820400 -1.34091600 2.12126800
H -1.52746700 2.91172300 -1.77254300
H -0.50125900 4.33149900 -1.57789200
H -2.14196400 3.61417700 0.33677400
H -0.56403700 4.23304900 0.77303100
H 1.52723700 3.46469700 2.47327800
H 2.46955400 2.05221600 2.06057600
H 3.04084200 3.56955400 0.36706000
H 1.43679400 4.26440400 0.24956500
Ir 0.06407900 0.36715600 -0.05748900
C 2.55318100 -0.33023400 0.78240000
C 2.65874300 -0.73561800 2.13716900
C 2.25032900 -1.99041400 2.53403500
C 1.71176900 -2.91232200 1.60700600
H 3.02632700 0.59723200 0.45204600
H 3.08806700 -0.04610200 2.87077900
H 2.34180300 -2.28009900 3.58430500
H 1.37340900 -3.89671000 1.94287500
O 2.03247700 -2.67983700 -1.93225200
H -0.52415000 -2.92559300 -2.34308600
H -0.05352800 -0.58073300 -2.54918900
H -0.99912100 -2.56987100 -0.66859900
C 1.11510300 -3.18949000 -0.97091300
H 1.09042100 -4.28771100 -0.98787500
C 2.73127300 -0.48538700 -2.57069400
H 3.79337500 -0.53926200 -2.28328200
H 2.62849700 -0.90329500 -3.58369400
H 2.42651100 0.56828700 -2.62212600

2f

Electronic energy at 338.15 K (1,4-dioxane): -1336.599637 au

Electronic energy + thermal free energy correction at 338.15 K (1,4-dioxane): -1336.196060 au

C -2.70631700 -0.05921200 -0.52634100
C -4.01557700 -0.04878200 -1.05357600
C -2.44599000 -0.84487600 0.61520000
C -5.00252800 -0.80584600 -0.44146400
H -4.22345300 0.55677300 -1.94018300
C -3.45700000 -1.59752600 1.22586300
C -4.73914800 -1.58426900 0.69884100
H -6.01444300 -0.79439800 -0.86003500
H -3.20490400 -2.18749500 2.11266100
H -5.53836500 -2.16976000 1.16113500
C -0.18026700 -2.47458900 -1.39680700
C 1.62438100 -2.54020600 0.29255700
C 2.06862800 -1.26123600 -0.13632700
C 1.86514900 -1.28983900 -1.64695600
C 0.34175700 -1.04504600 -1.62928500
H 0.47041500 1.90252000 -2.47429700
C 0.51983100 2.45906700 -1.52881900
C -0.67653700 3.30544300 -1.20036900
C 1.68699200 2.45189200 -0.84212400
C 1.62258200 2.66525600 1.71198700
C 1.97011900 3.30623700 0.36147000
H 2.52449000 1.87894300 -1.25279600
C 0.37329900 1.82727600 1.75504900
C -1.06699800 3.37889900 0.28602200
C -0.82062300 2.14444200 1.12811800
H 0.31989900 1.12267700 2.59585000
